# Supplementary material for: NOTCH and AKT Signalling Interact to Drive Mammary Tumour Heterogeneity
Source: Cancers (Basel). 2023 Aug 29;15(17):4324. doi: 10.3390/cancers15174324 (PMC10486941; doi:10.3390/cancers15174324)
Supplement: Supplementary file 1 [file cancers-15-04324-s001.zip › Supplementary Figure S2_revison 1.pdf]

GEL SET 1 QUANTIFIED BANDS

Gel 1

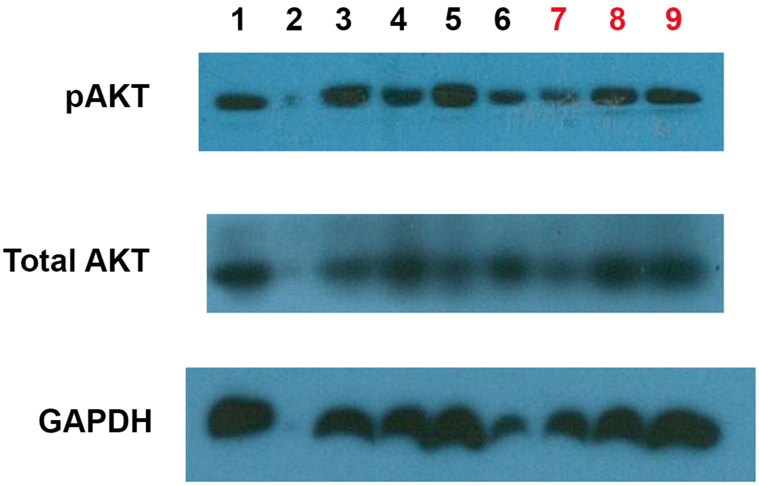

Gel 1.2

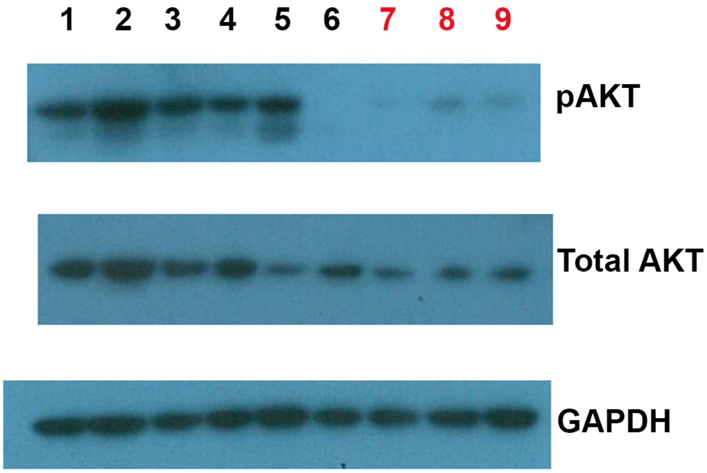

Gel 2.2

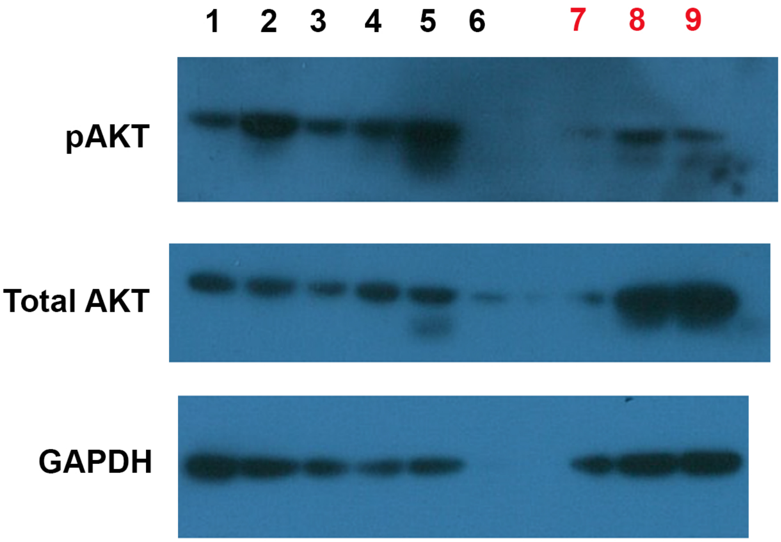

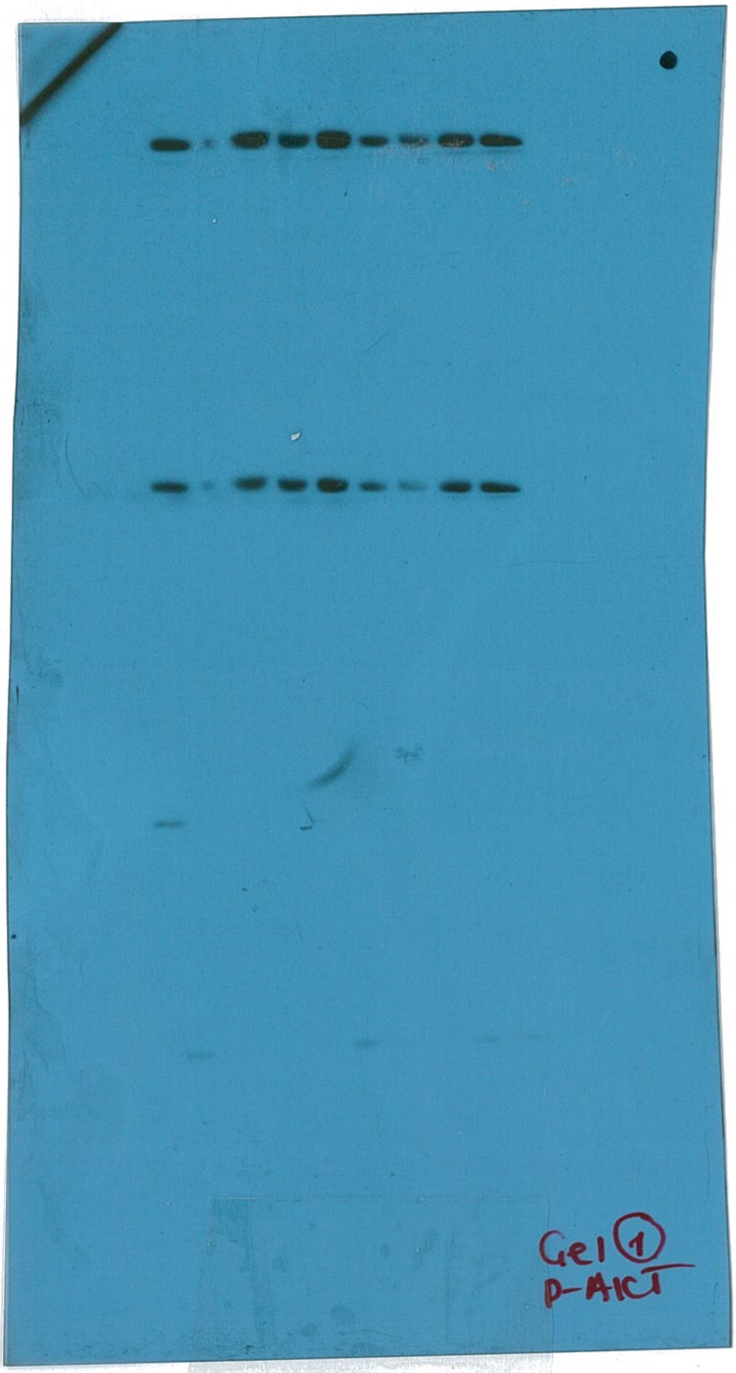
Gel 1
P-AKT

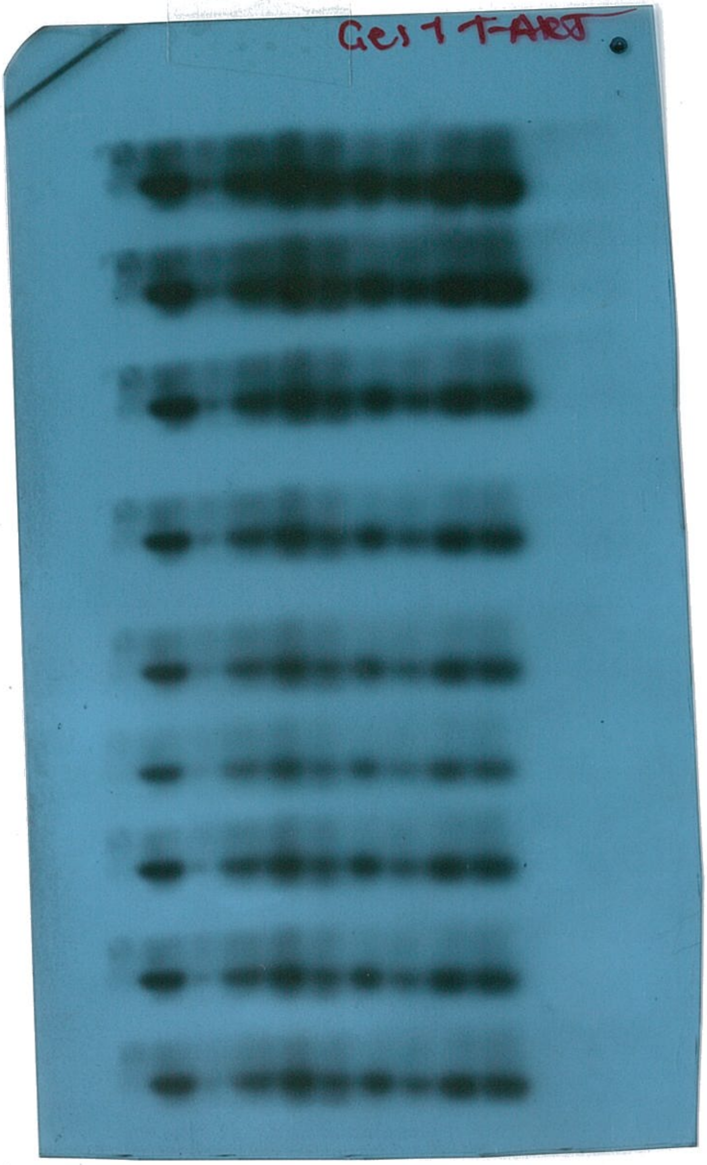
Gel 1 T-AKT

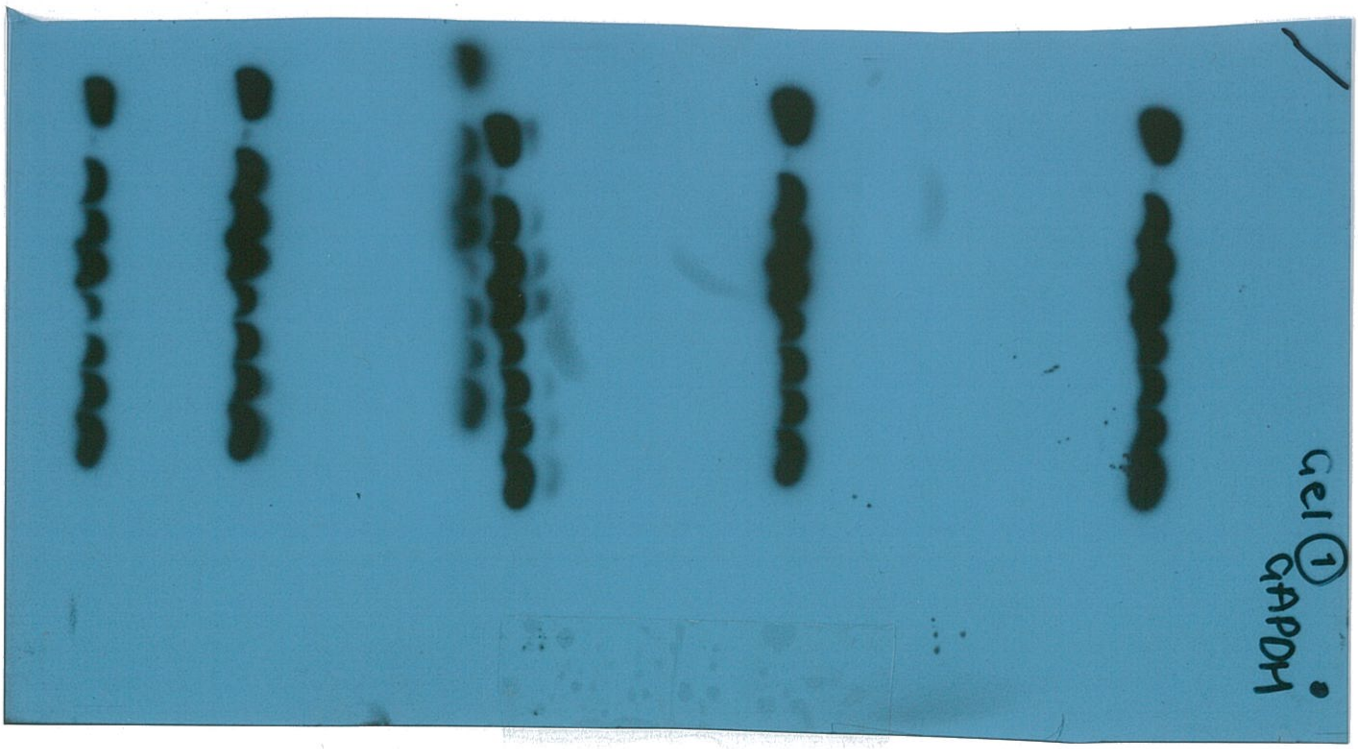
Gel 1
GAPDH

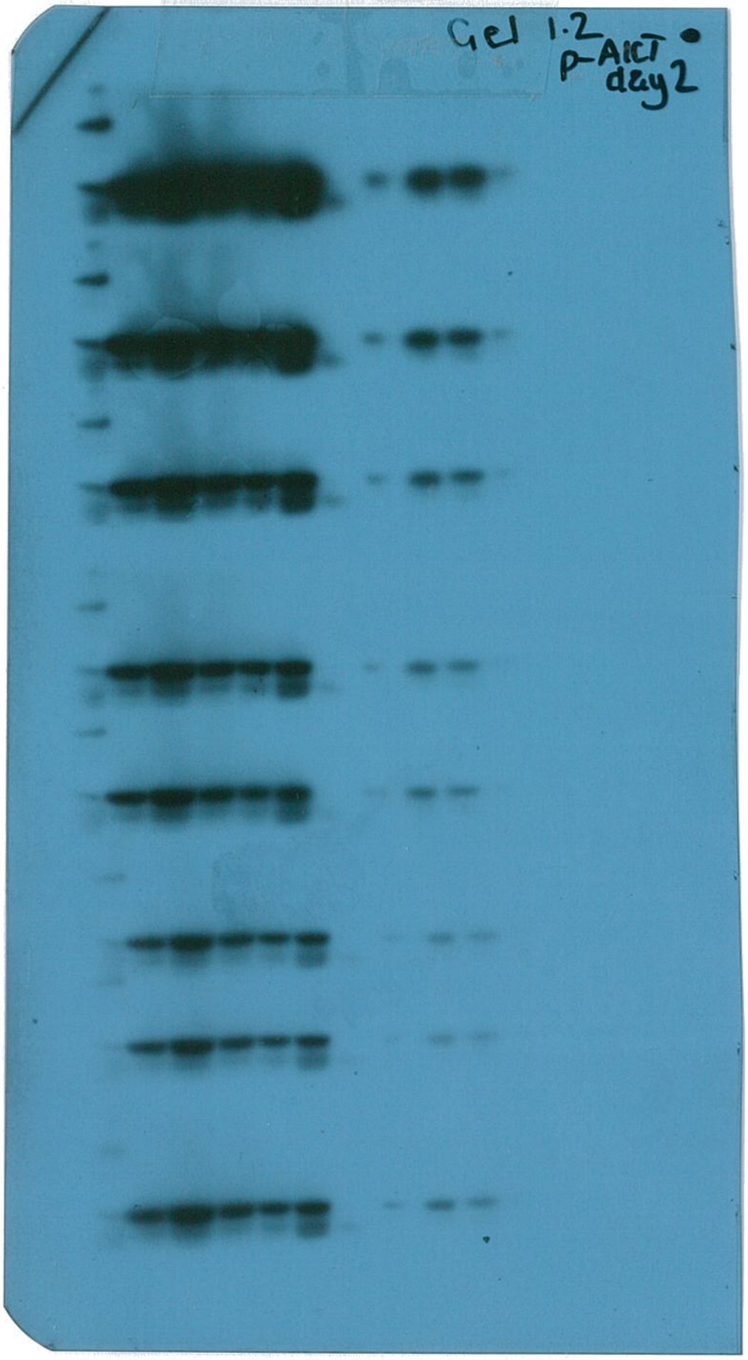
Gel 1.2
p-AKT
day2
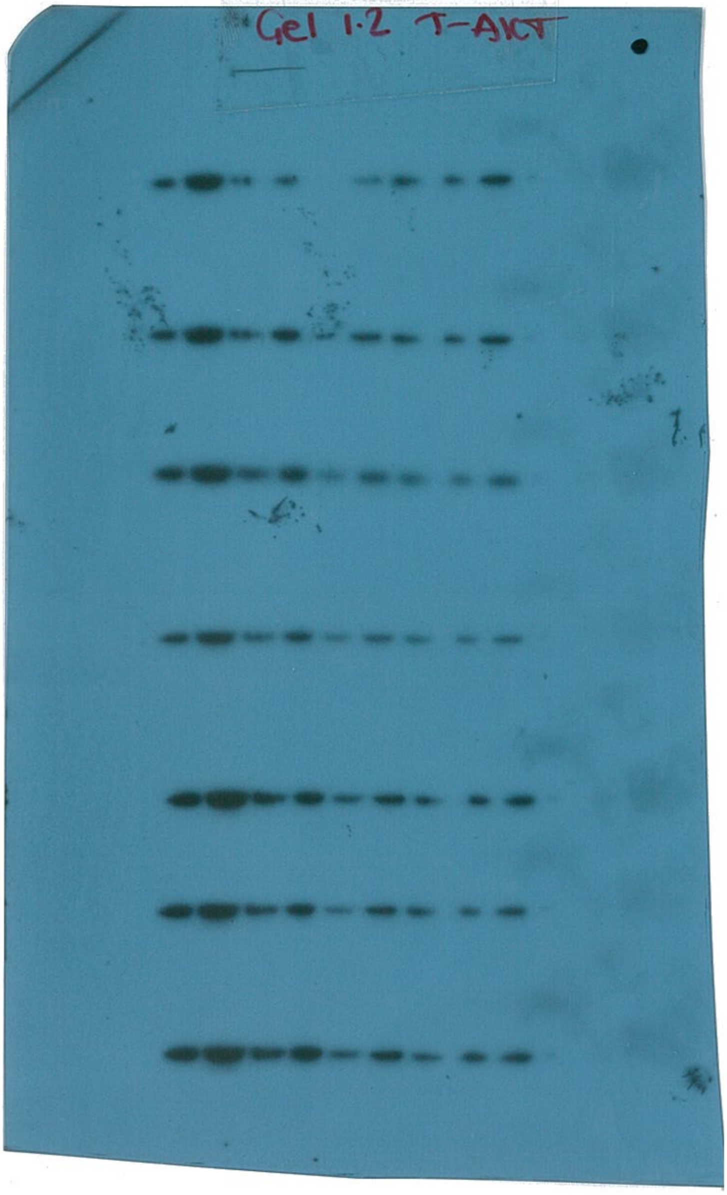
Gel 1.2 T-AKT
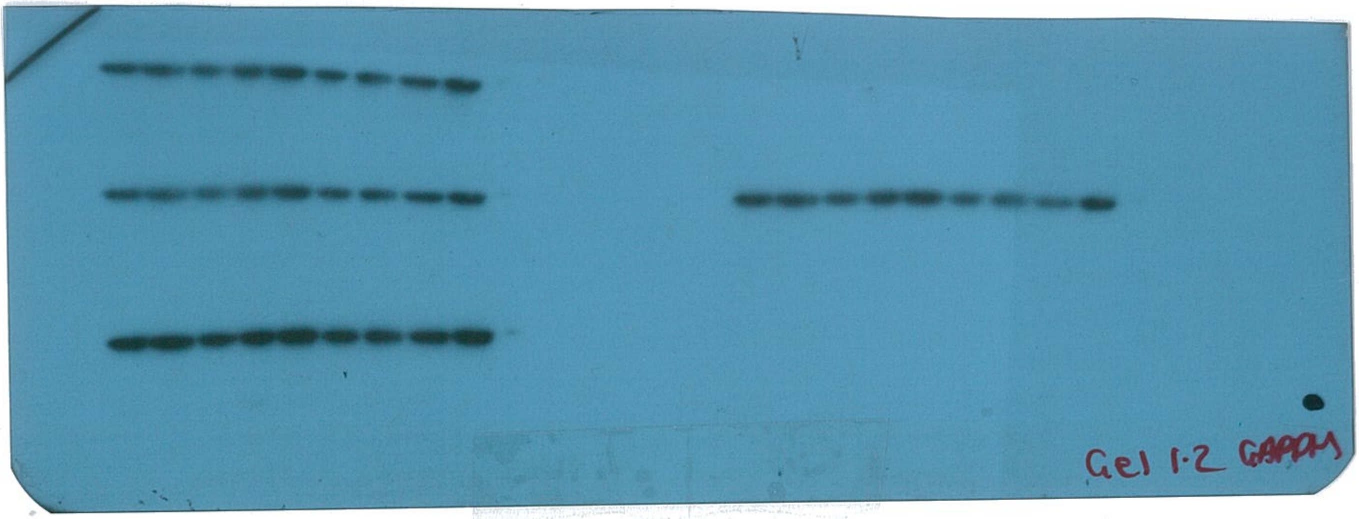
Gel 1.2 GAPDH

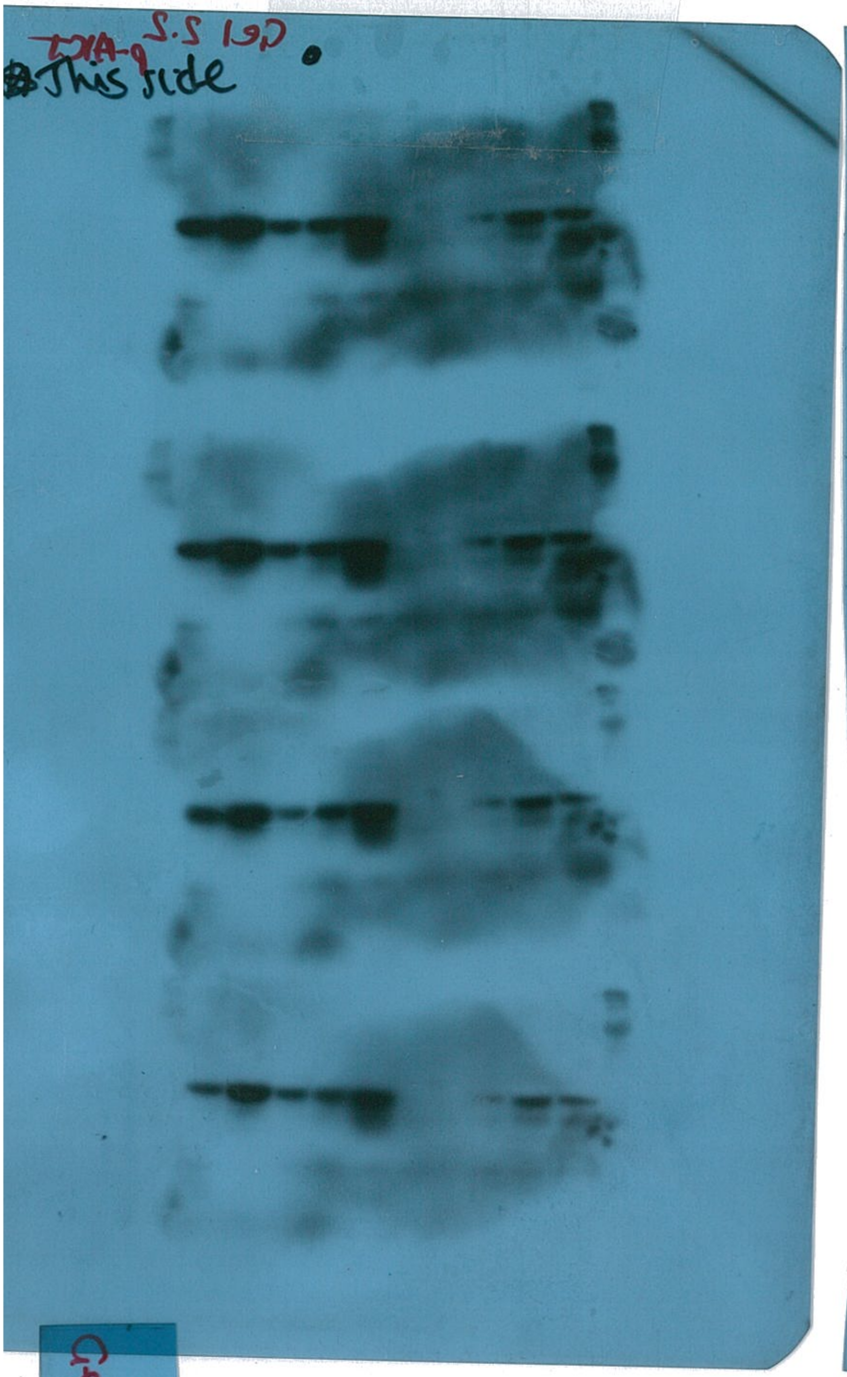
S.S 19D
This side

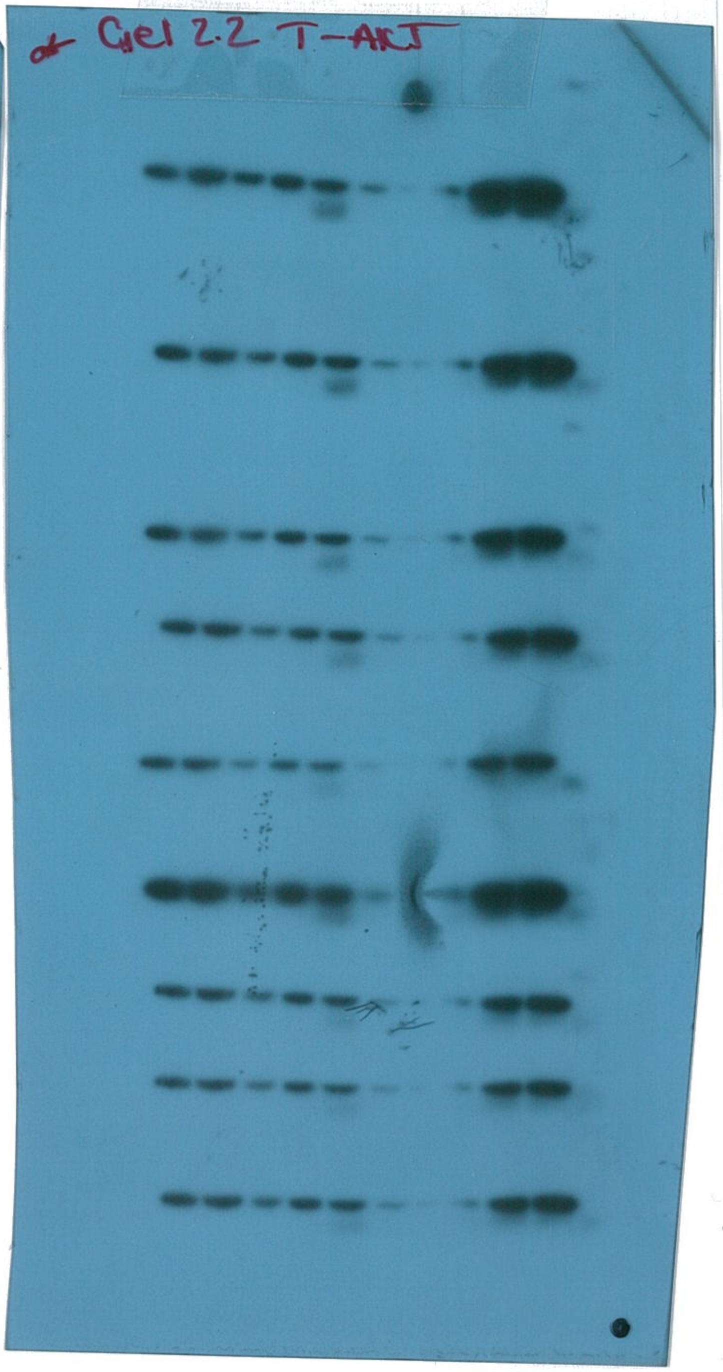
Gel 2.2 T-AKT

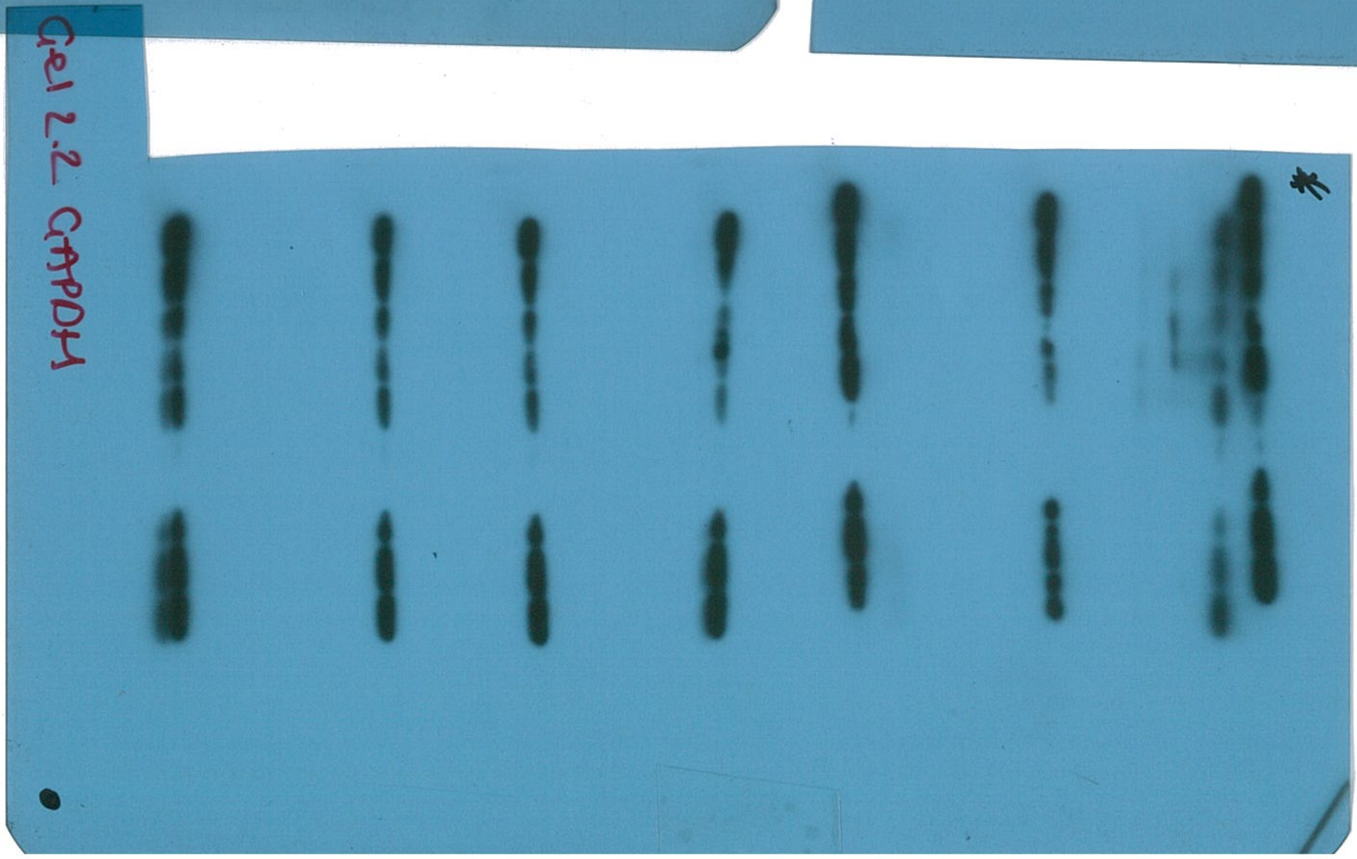
Gel 2.2 GAPDH

GEL SET 2 QUANTIFIED BANDS

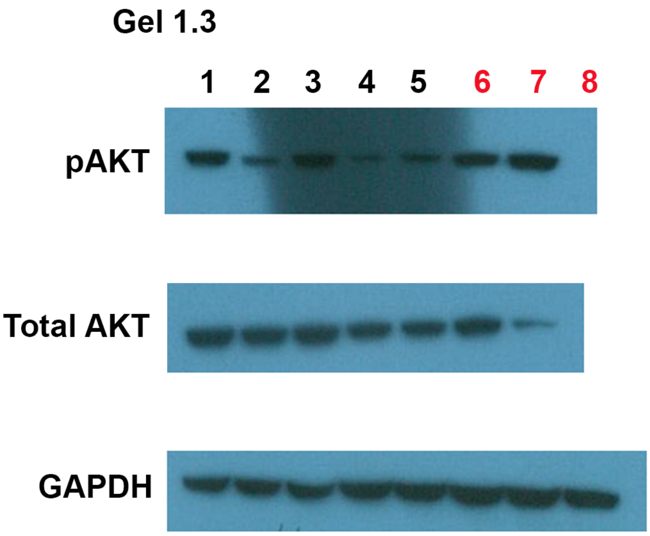

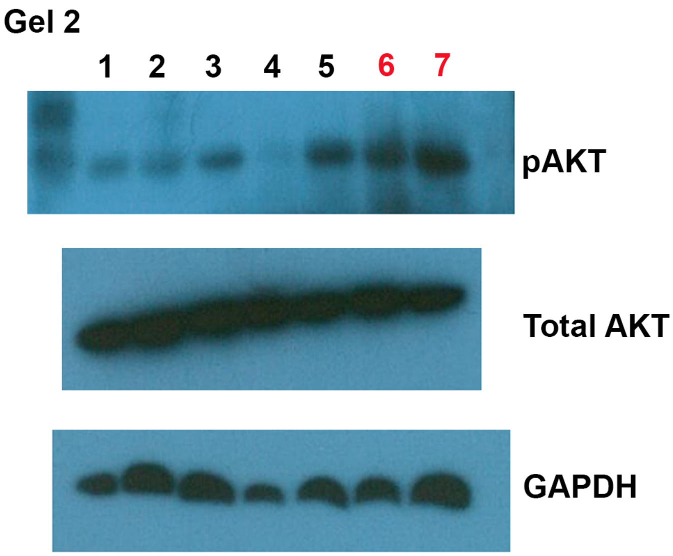

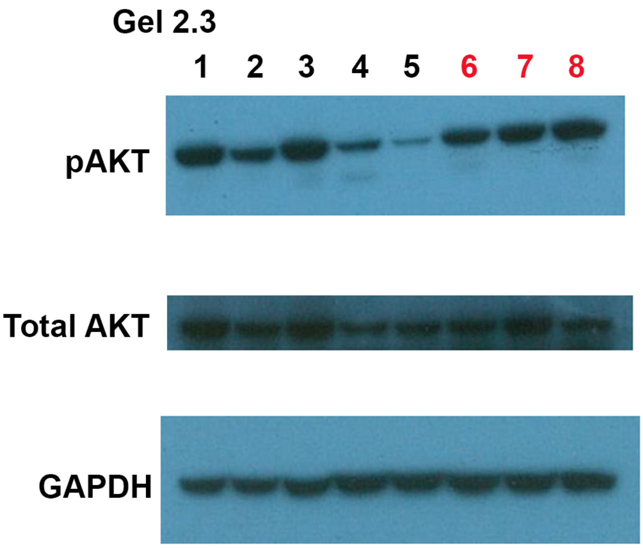

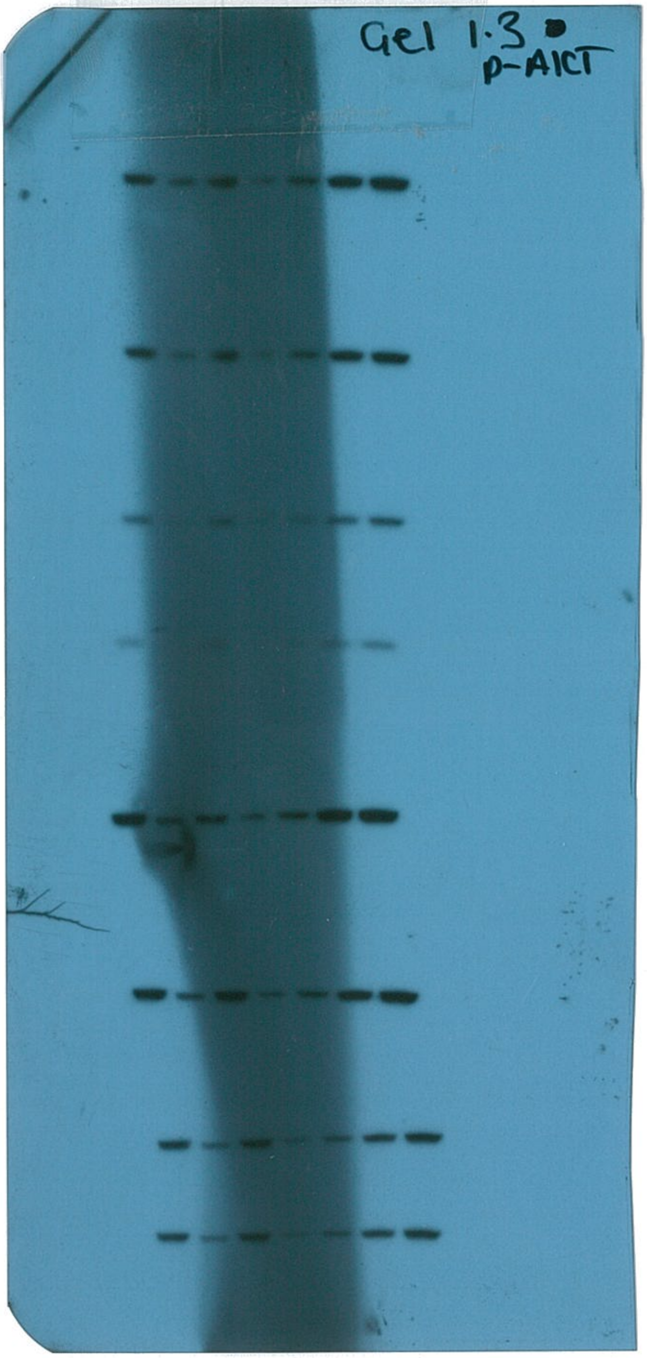
Gel 1.3
p-AKT

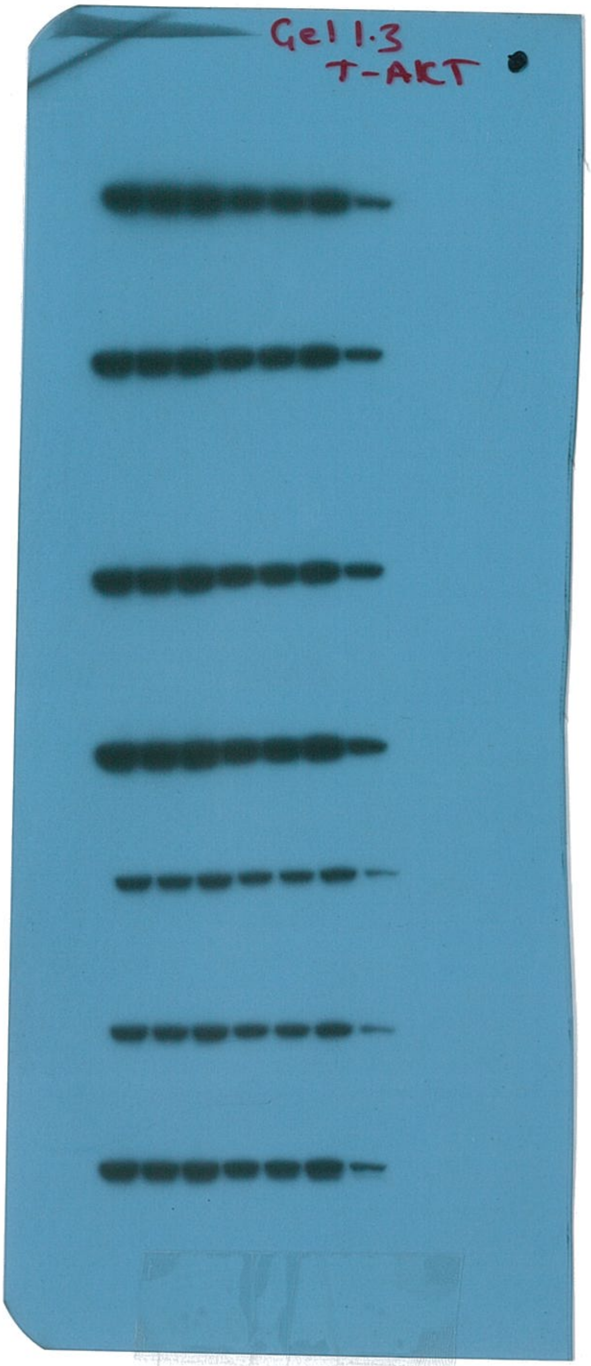
Gel 1.3
T-AKT

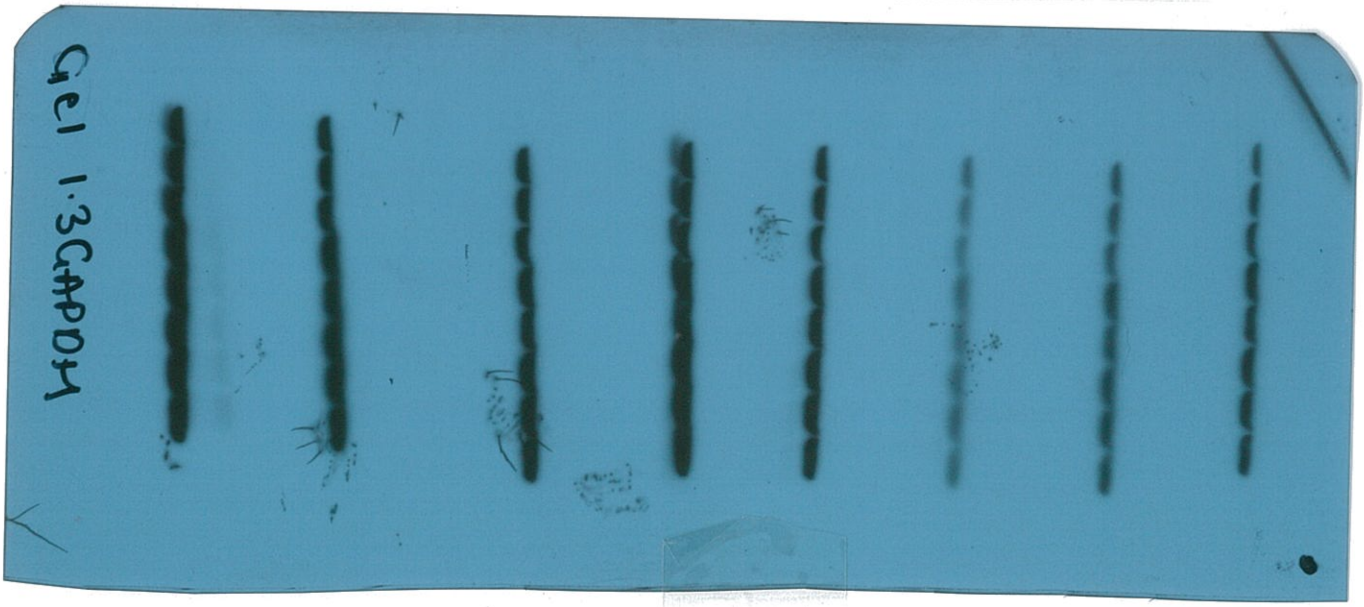
Gel 1.3GAPDH

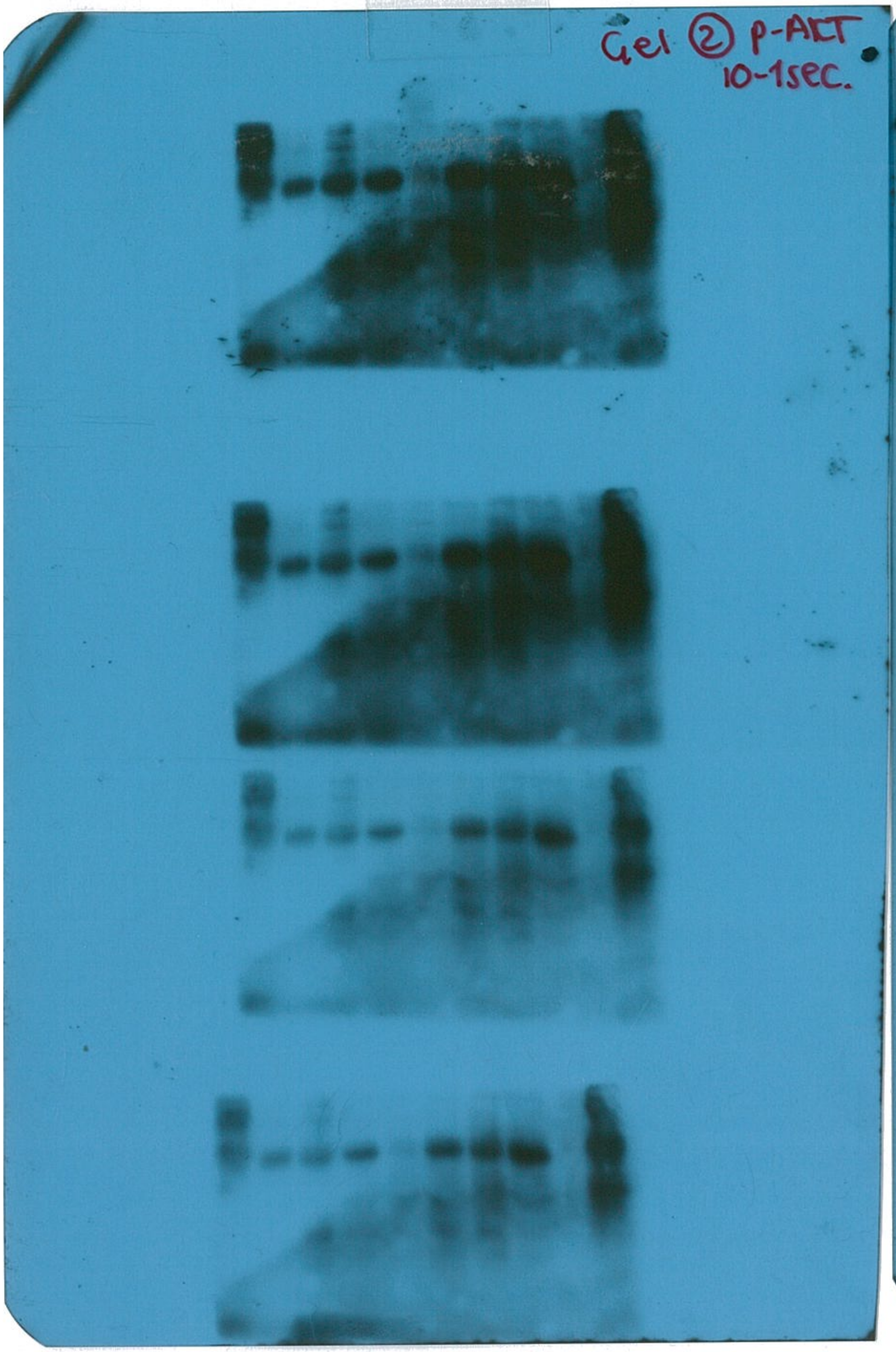
Gel ② p-AKT
10-1sec.

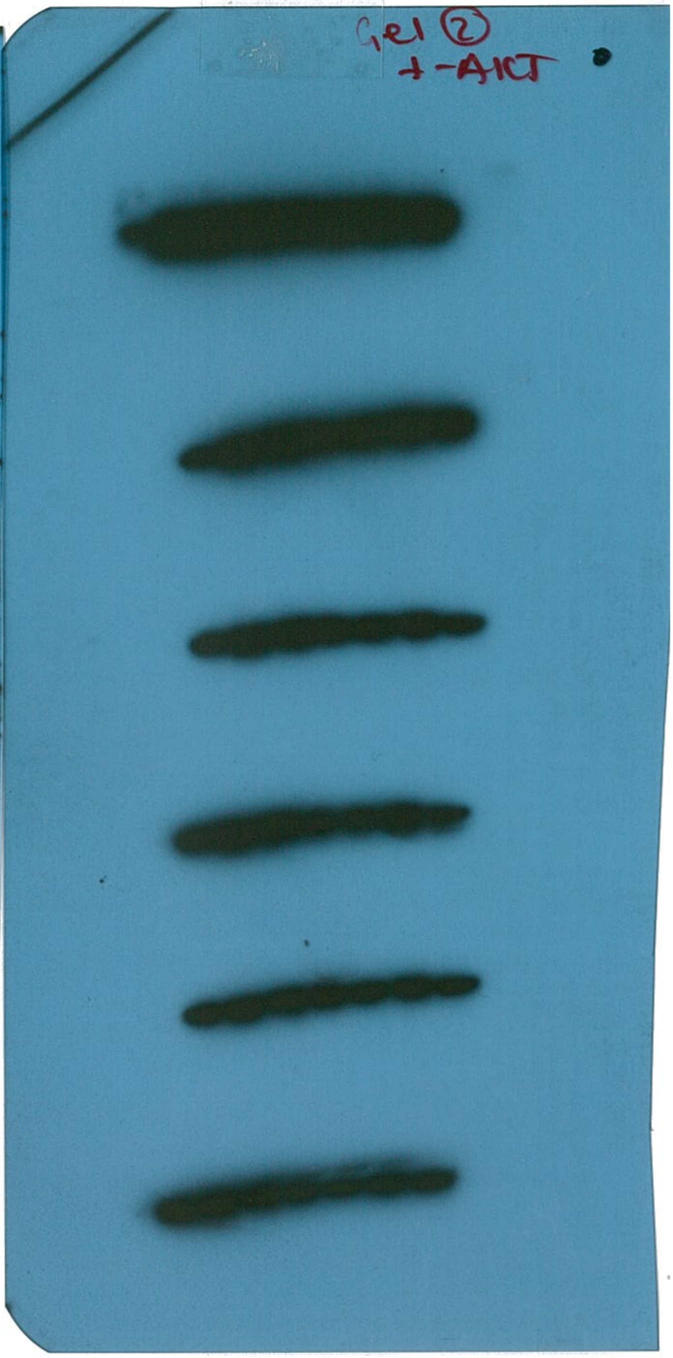
Gel ②
t-AKT

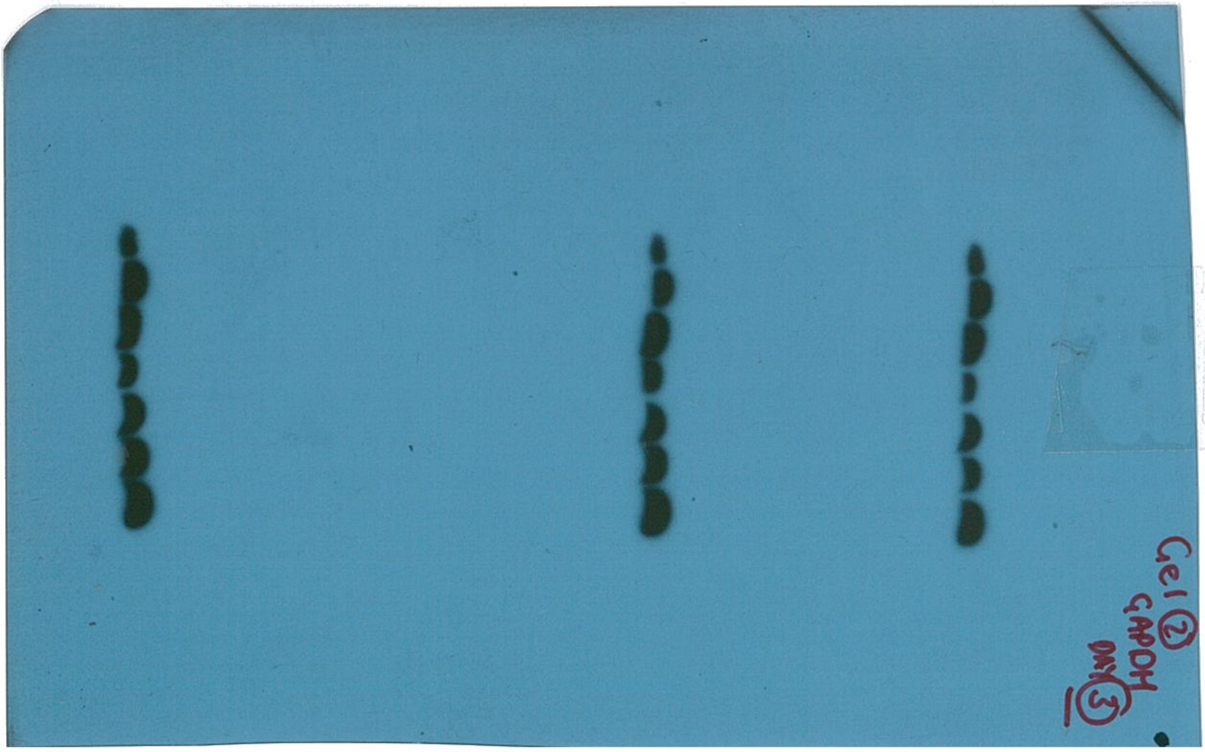
Gel ②
GAPDH
ONLY ③

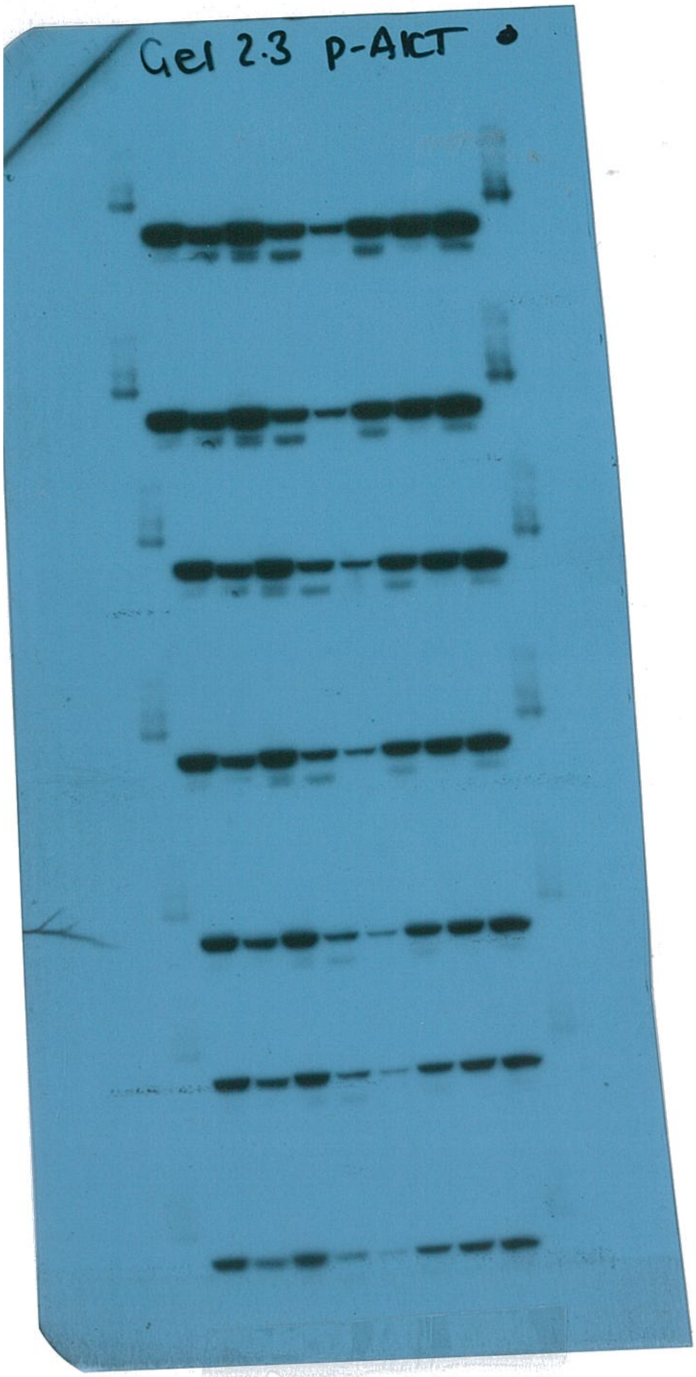
Gel 2.3 P-AKT

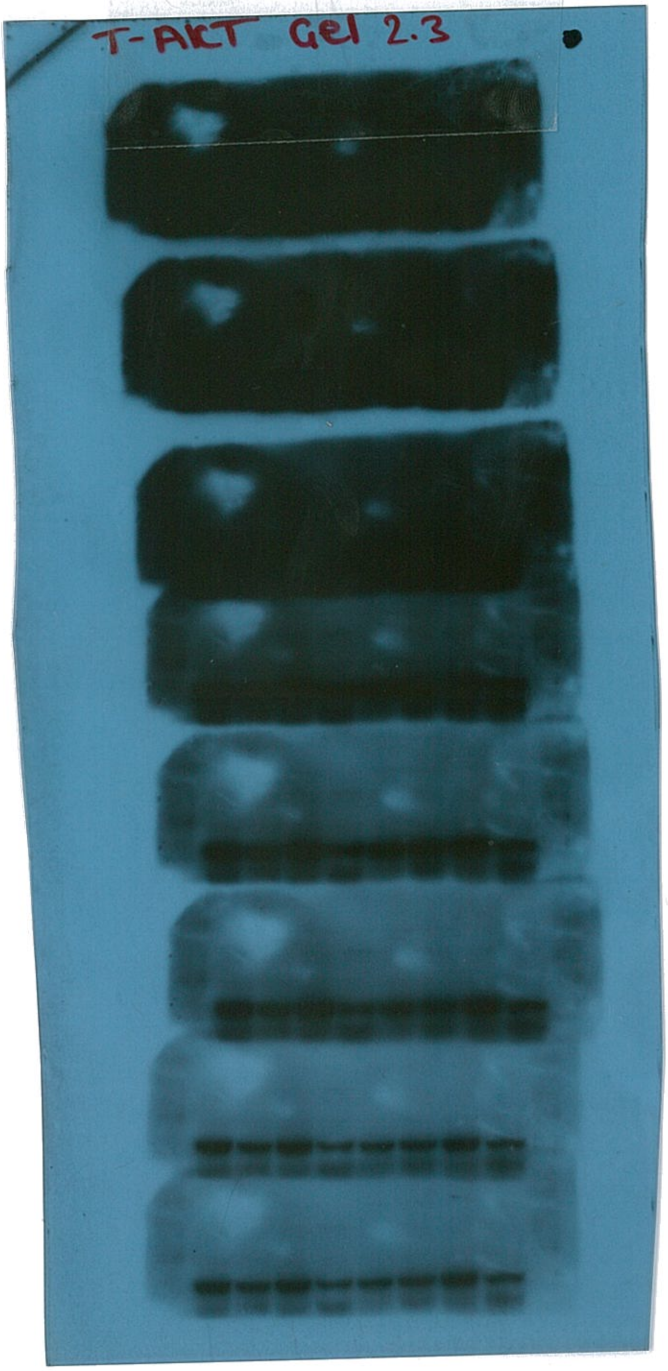
T-AKT Gel 2.3

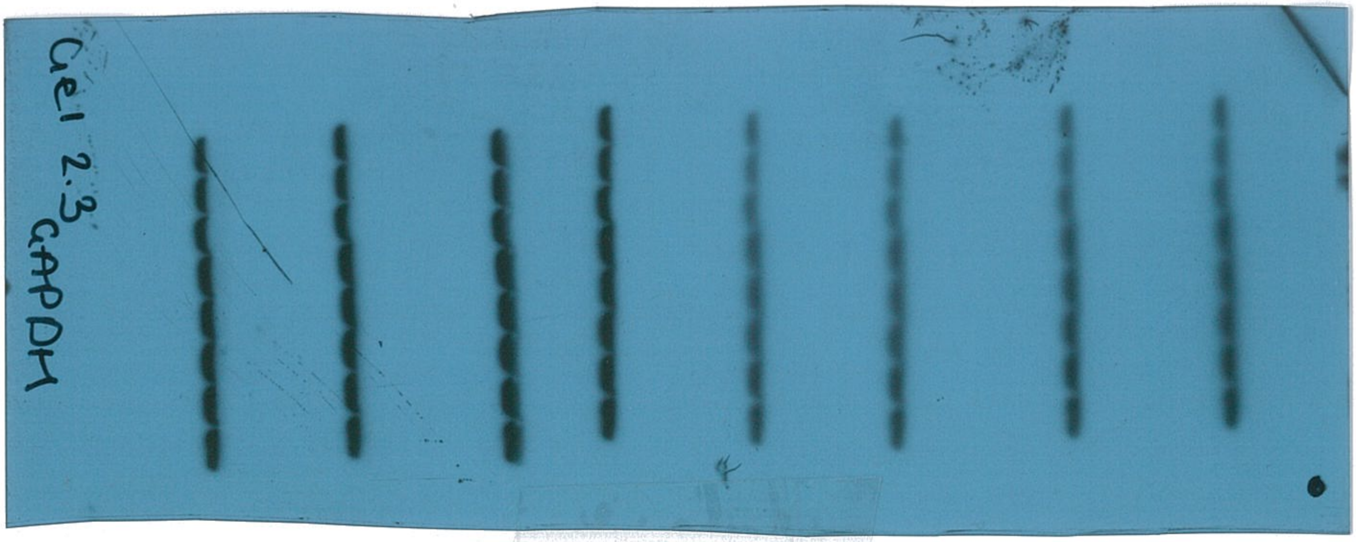
Gel 2.3
GAPDH

GEL SET 3 QUANTIFIED BANDS

Gel 1.4

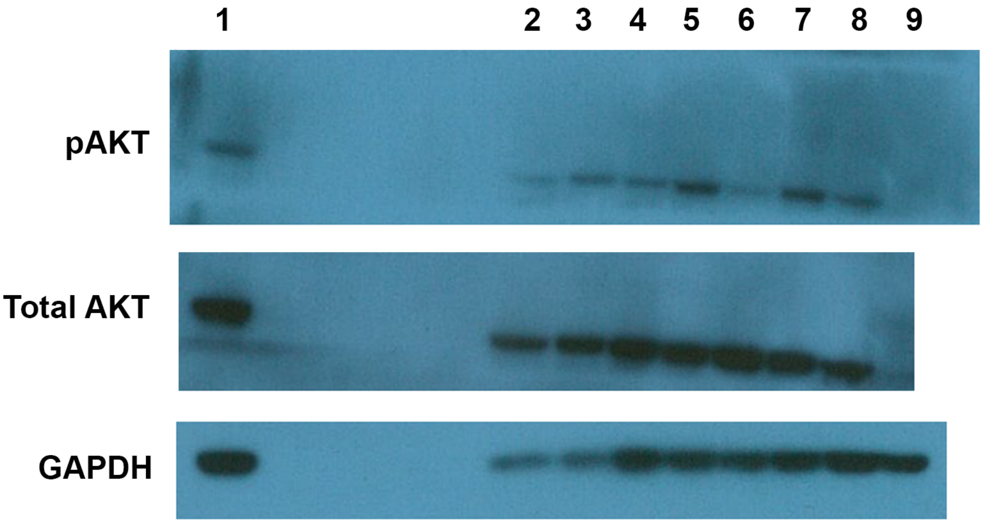

Gel 1.5

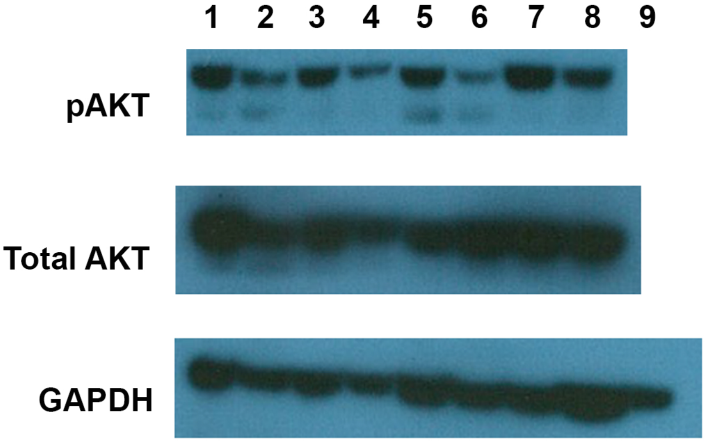

Gel 2.5

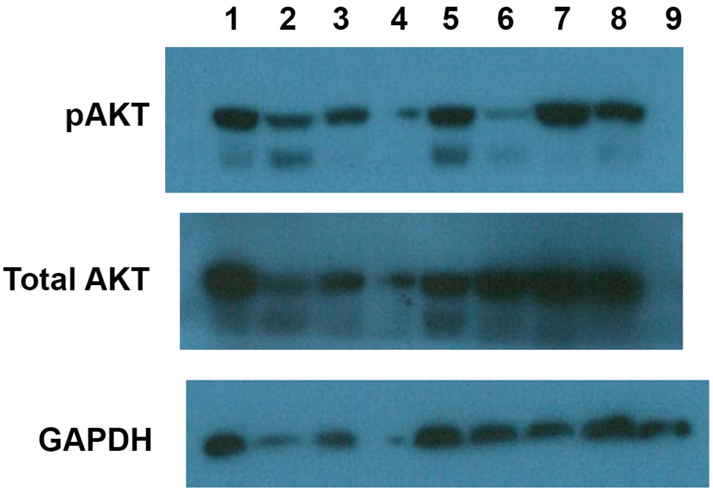

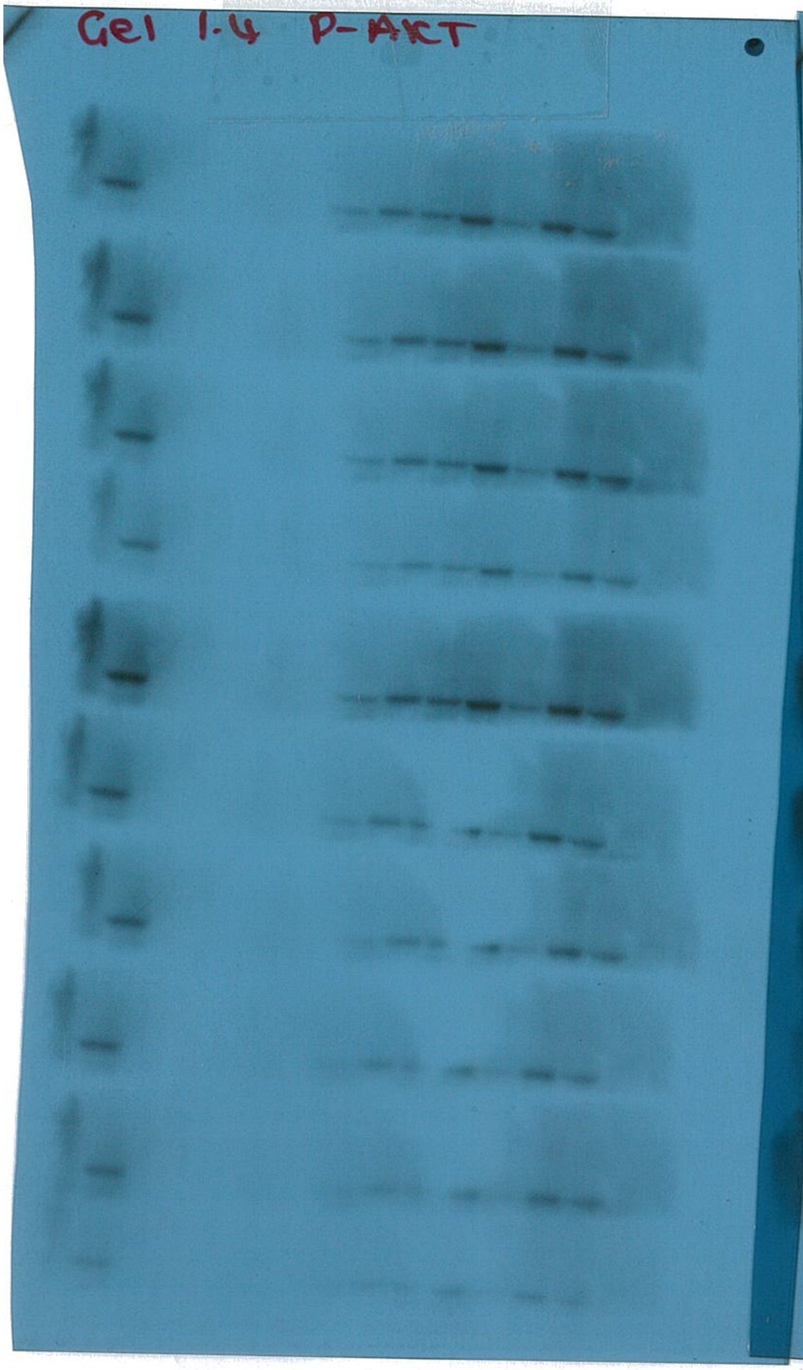
Gel 1.4 P-AKT

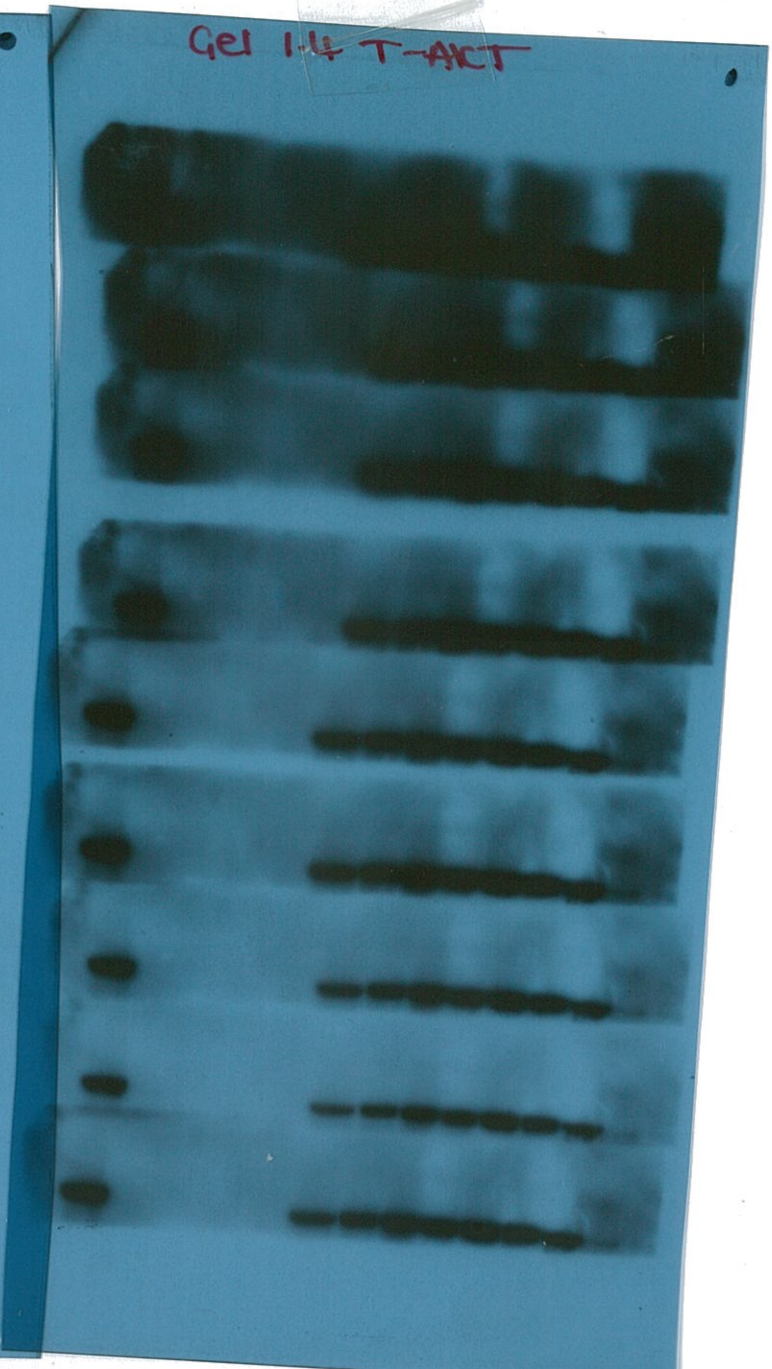
Gel 1.4 T-AKT

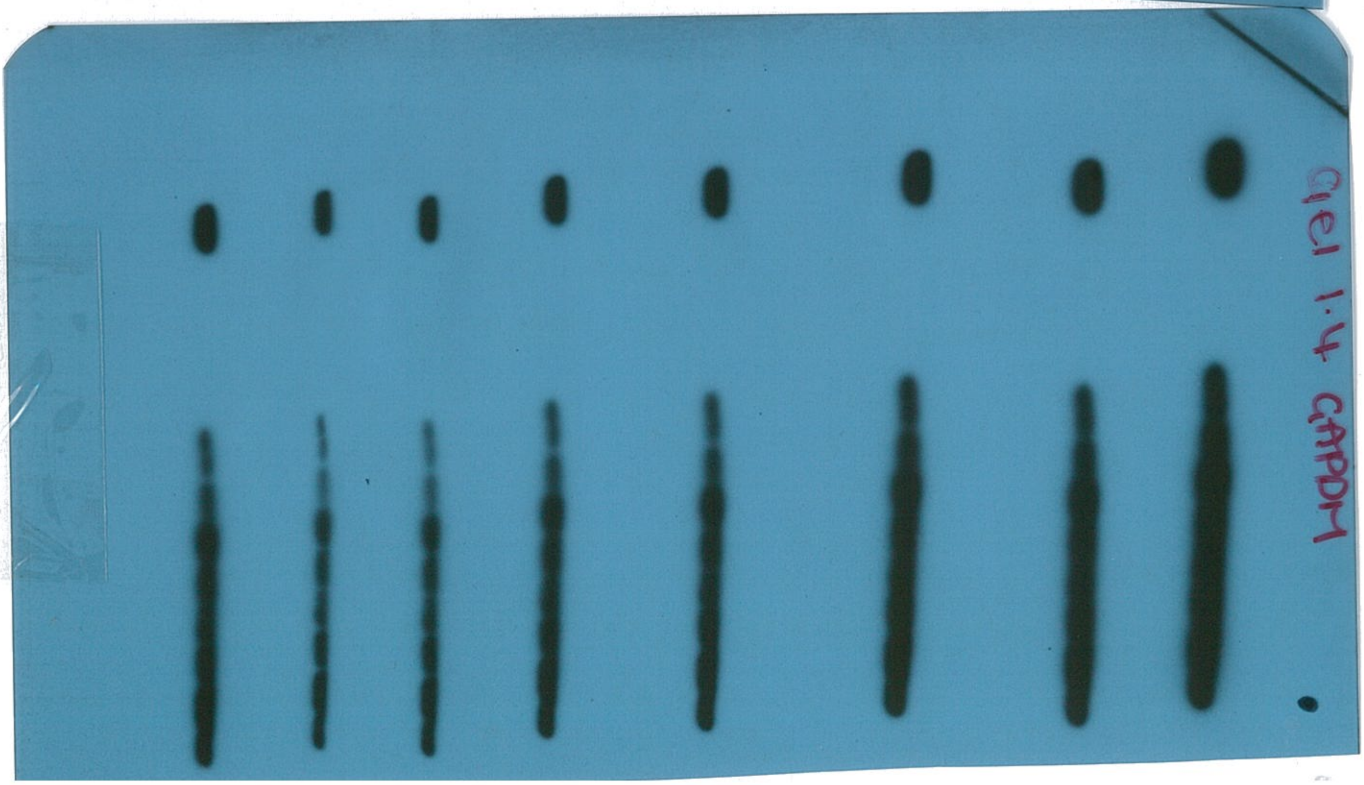
Gel 1.4 GAPDH

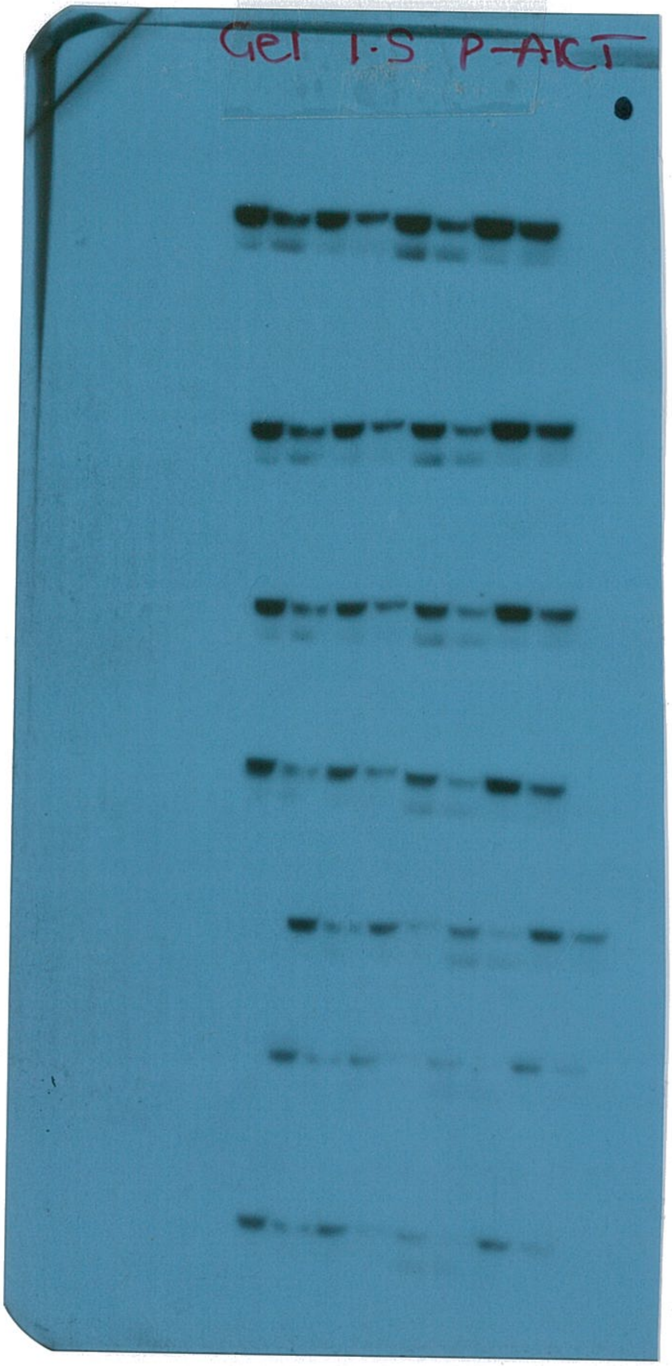
Gel 1.5 P-AKT

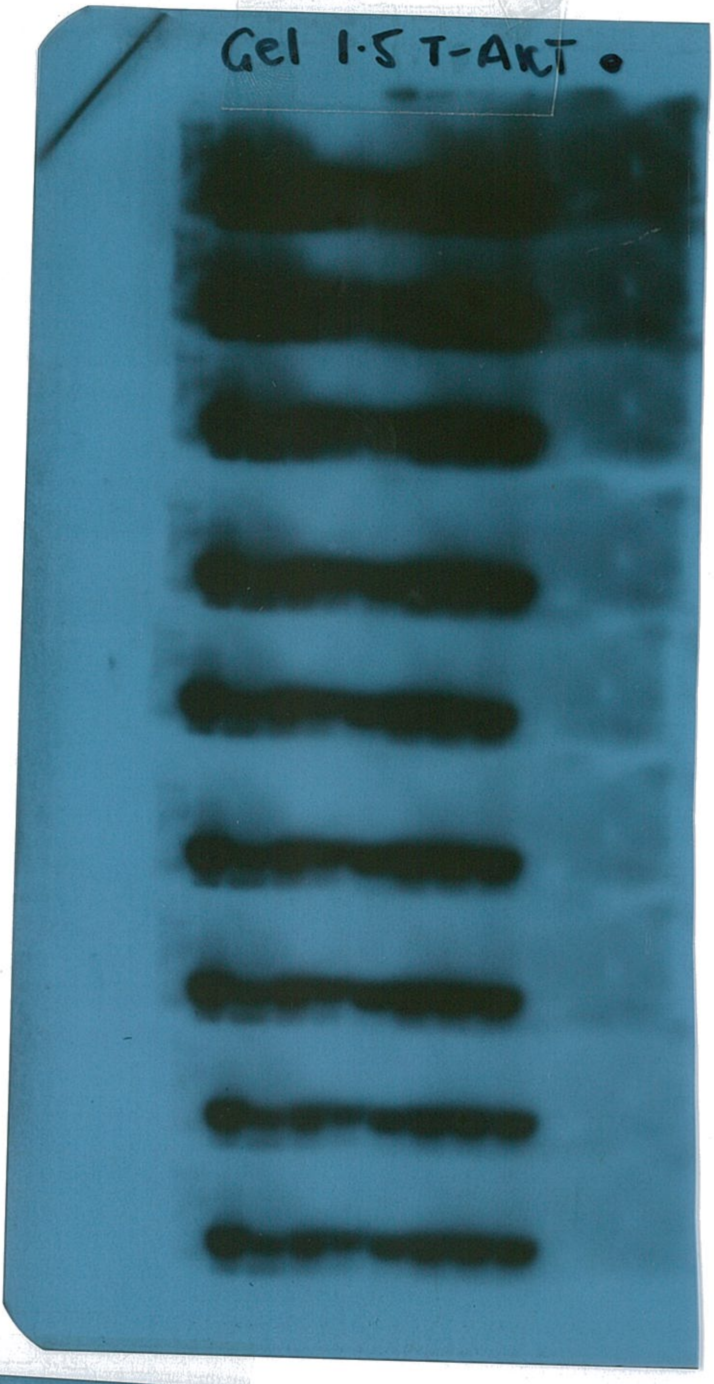
Gel 1.5 T-AKT

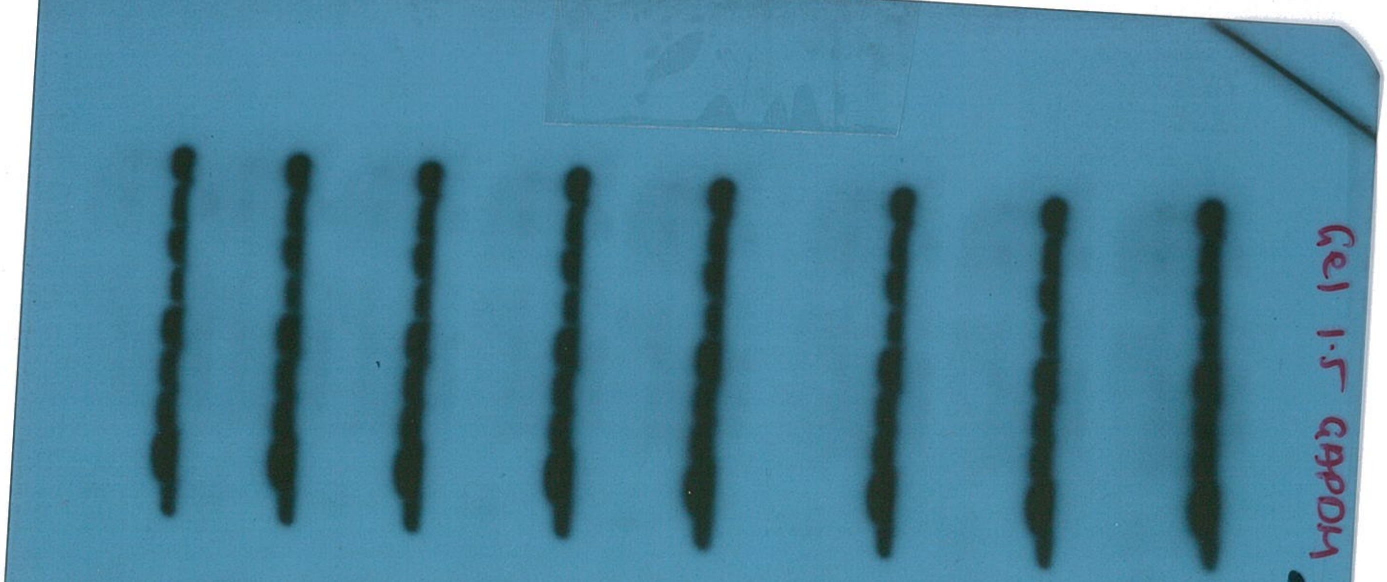
Gel 1.5 GAPDH

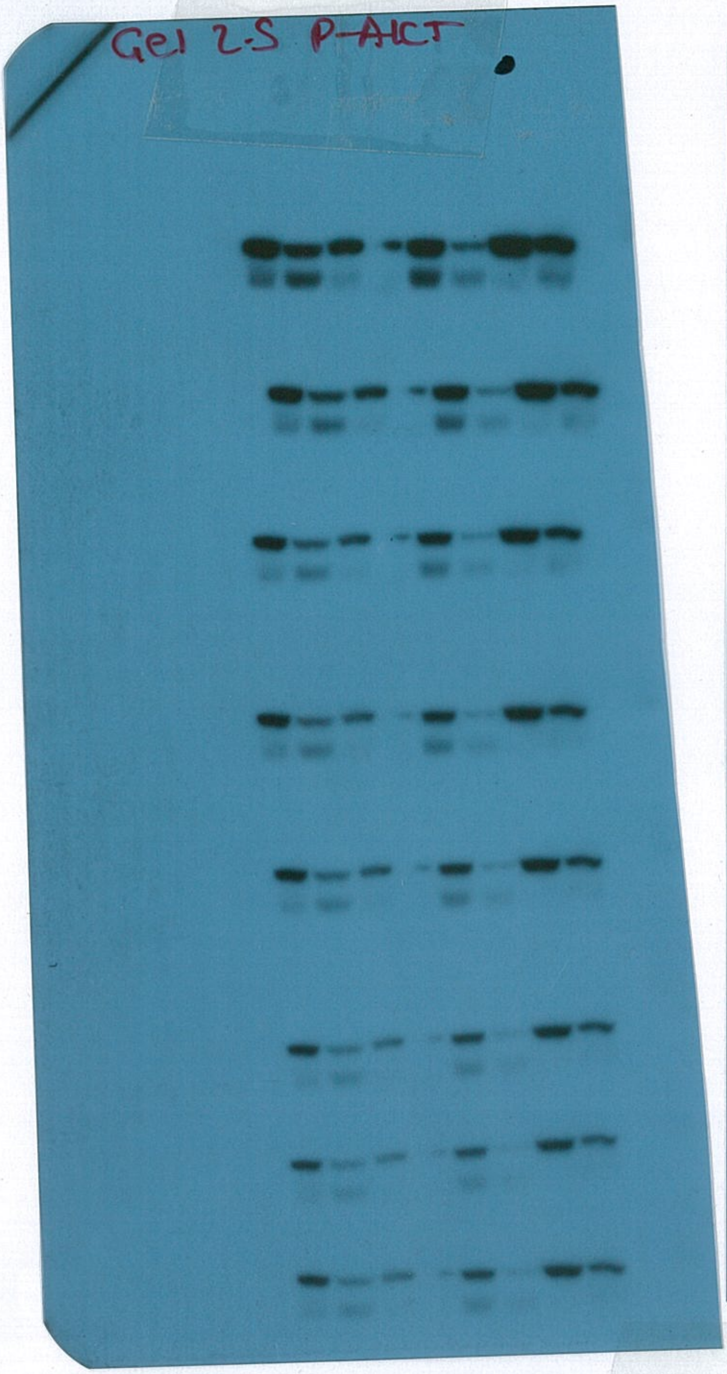
Gel 2.5 P-AKT

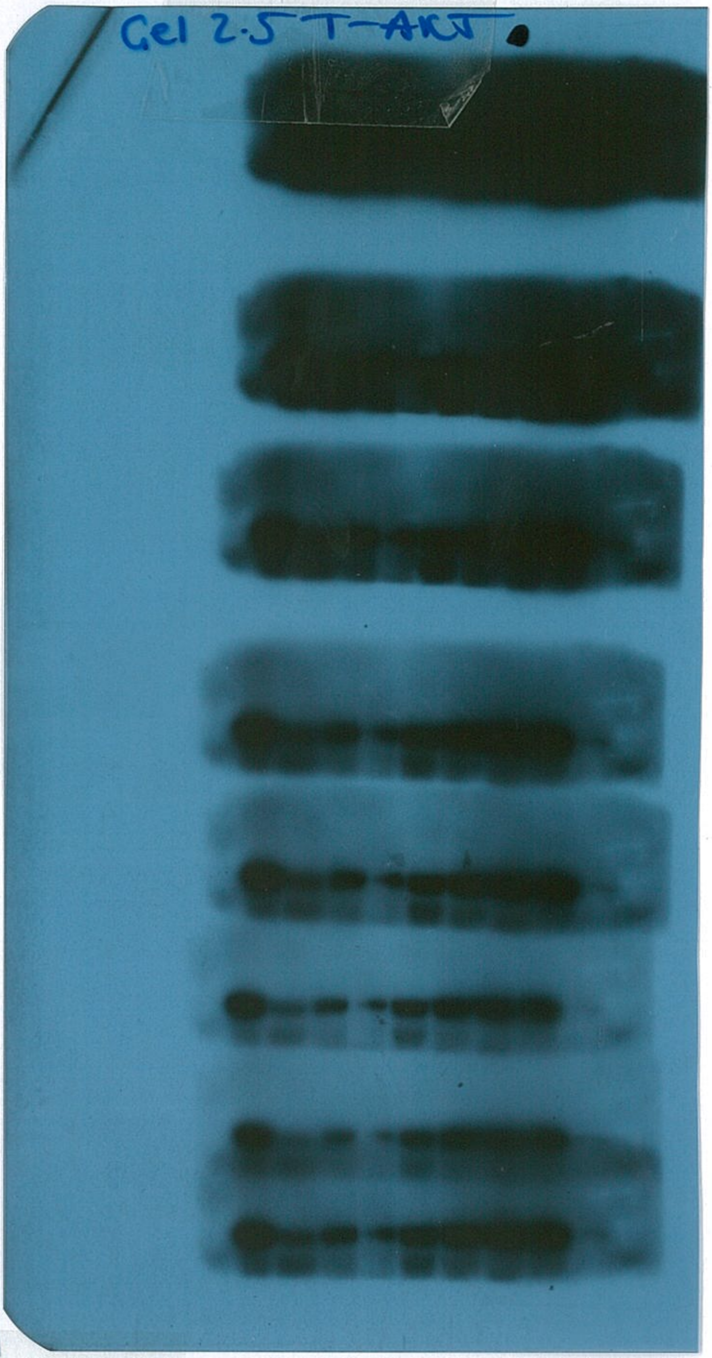
Gel 2.5 T-AKT

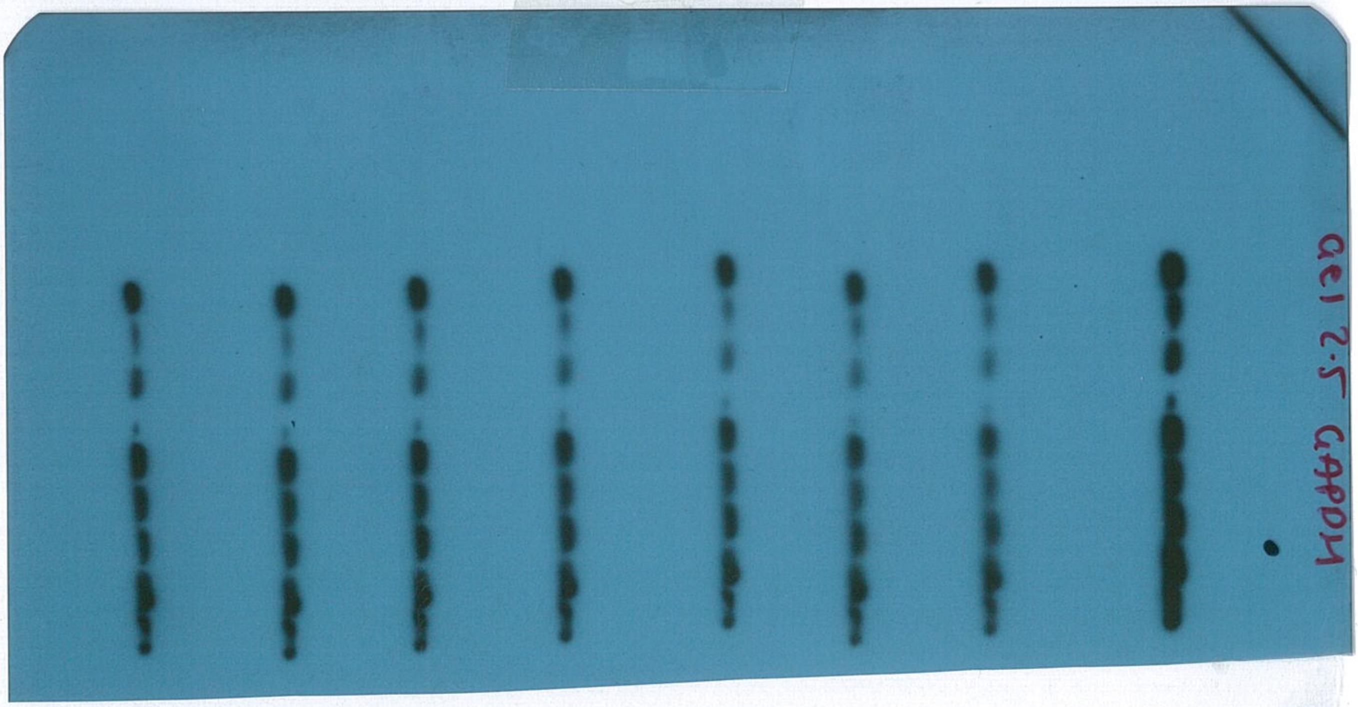
Gel 2.5 GAPDH

GEL SET 4 QUANTIFIED BANDS

Gel 1.6

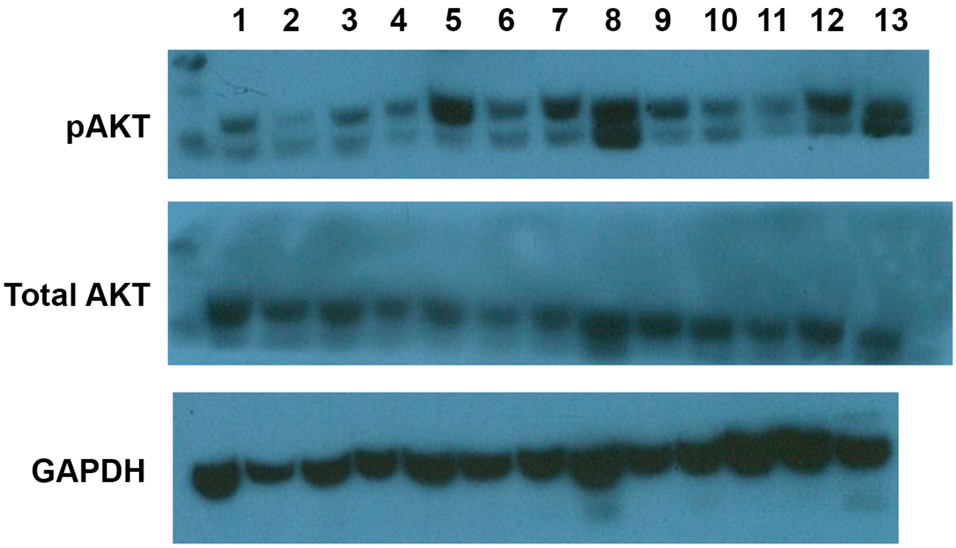

Gel 2.4

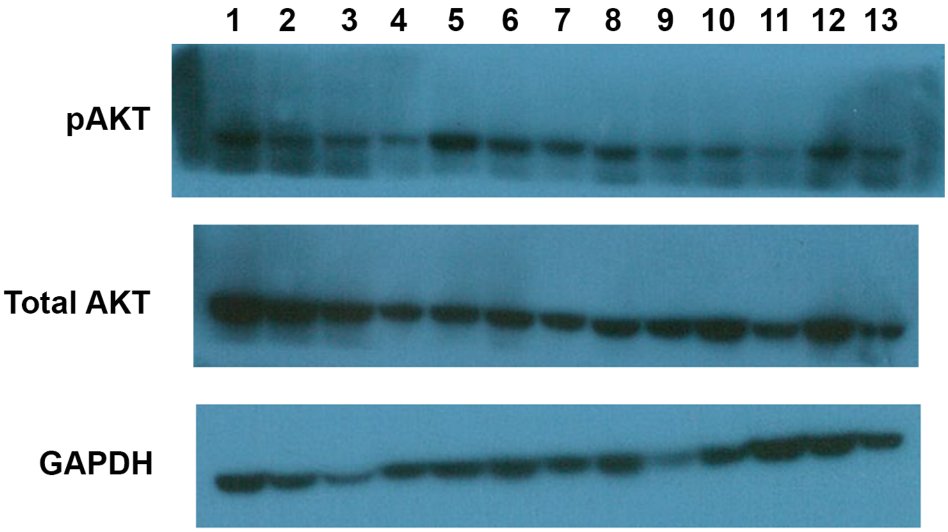

Gel 2.6

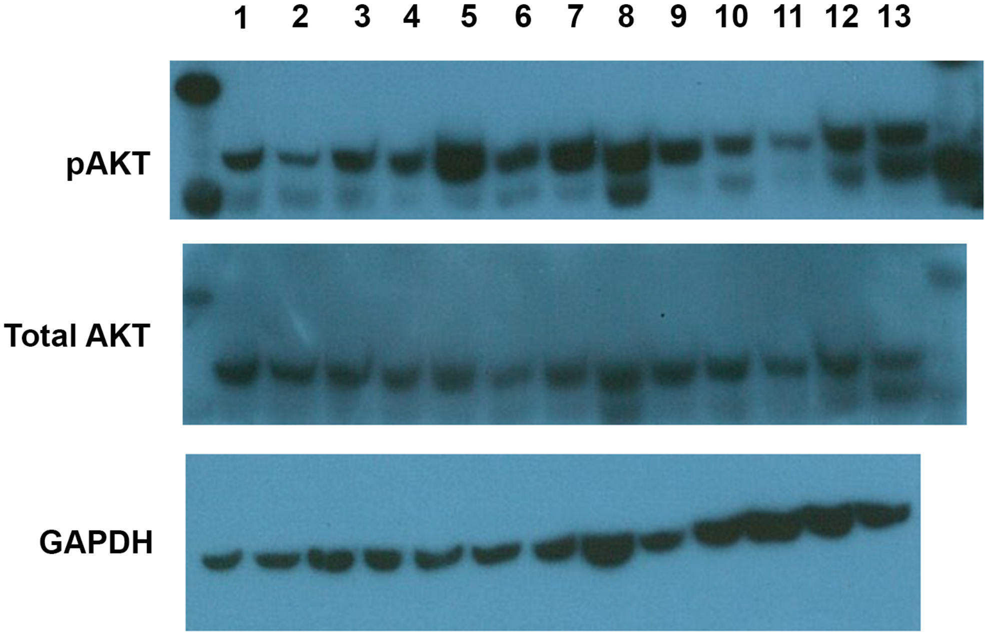

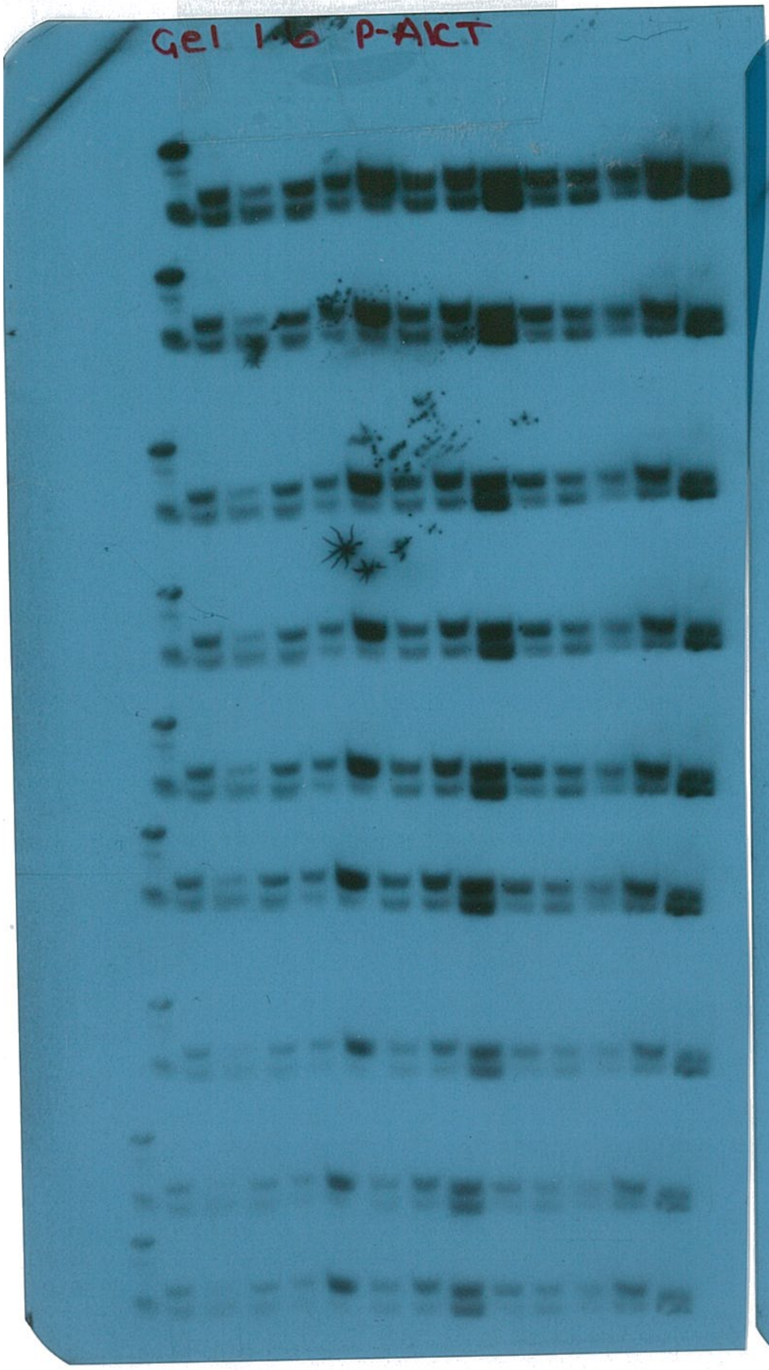
Gel 1.6 P-AKT

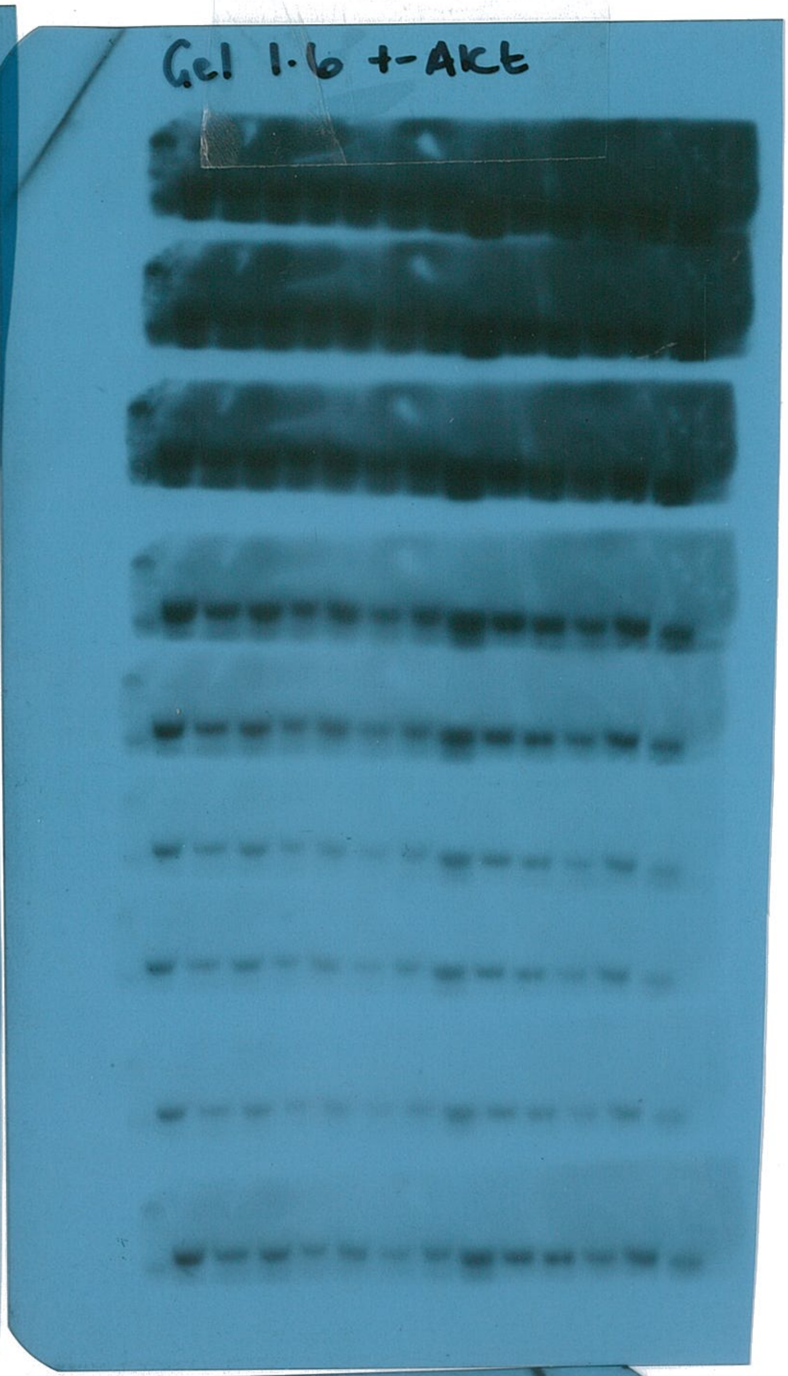
Gel 1.6 t-AKt

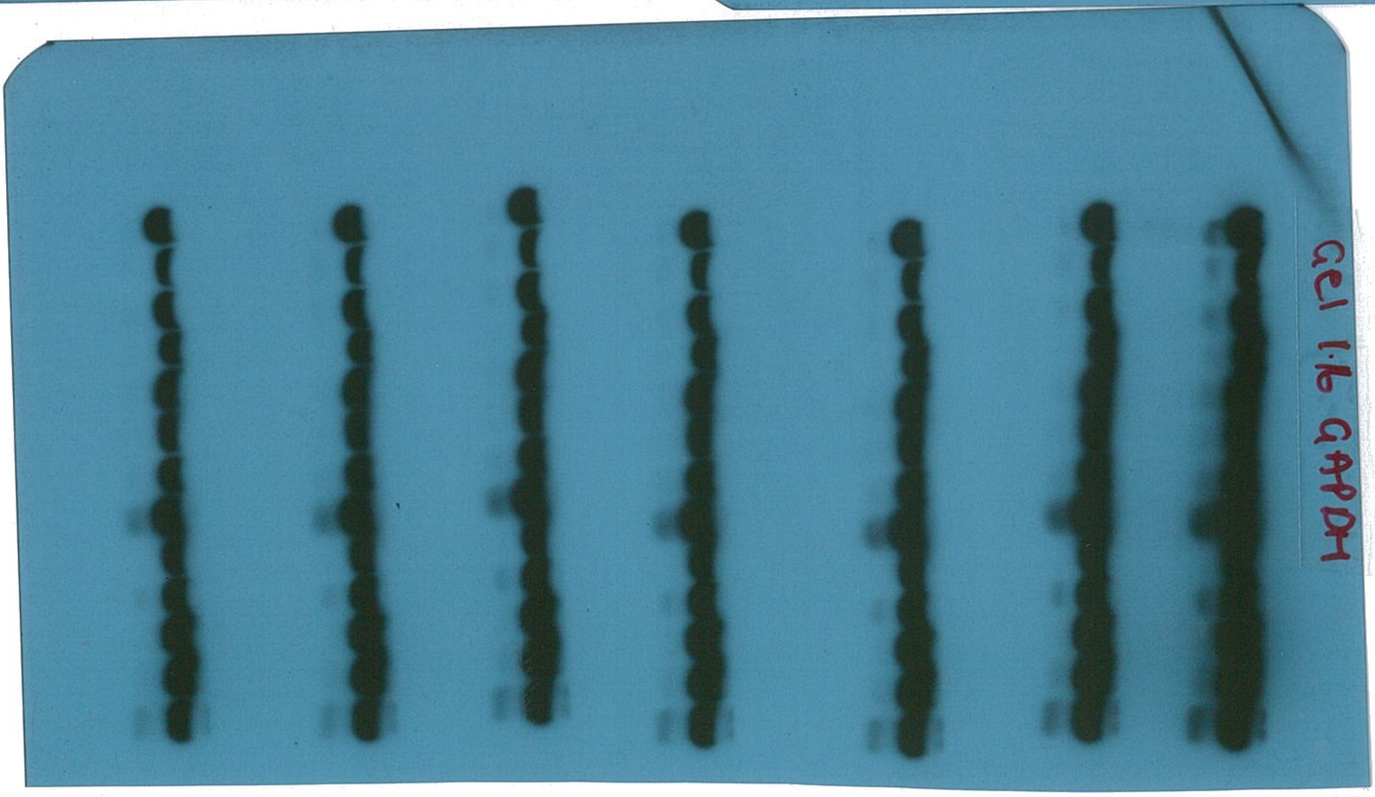
Gel 1.6 GAPDH

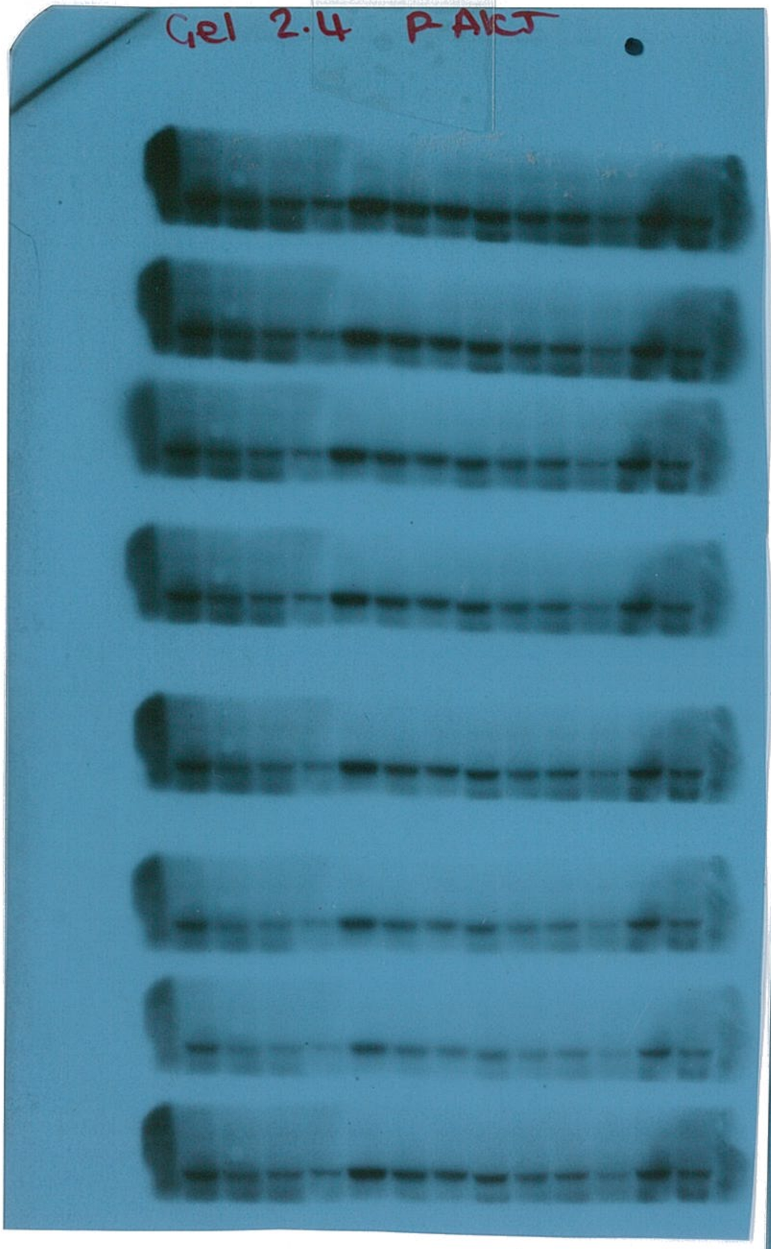
Gel 2.4 P-AKT

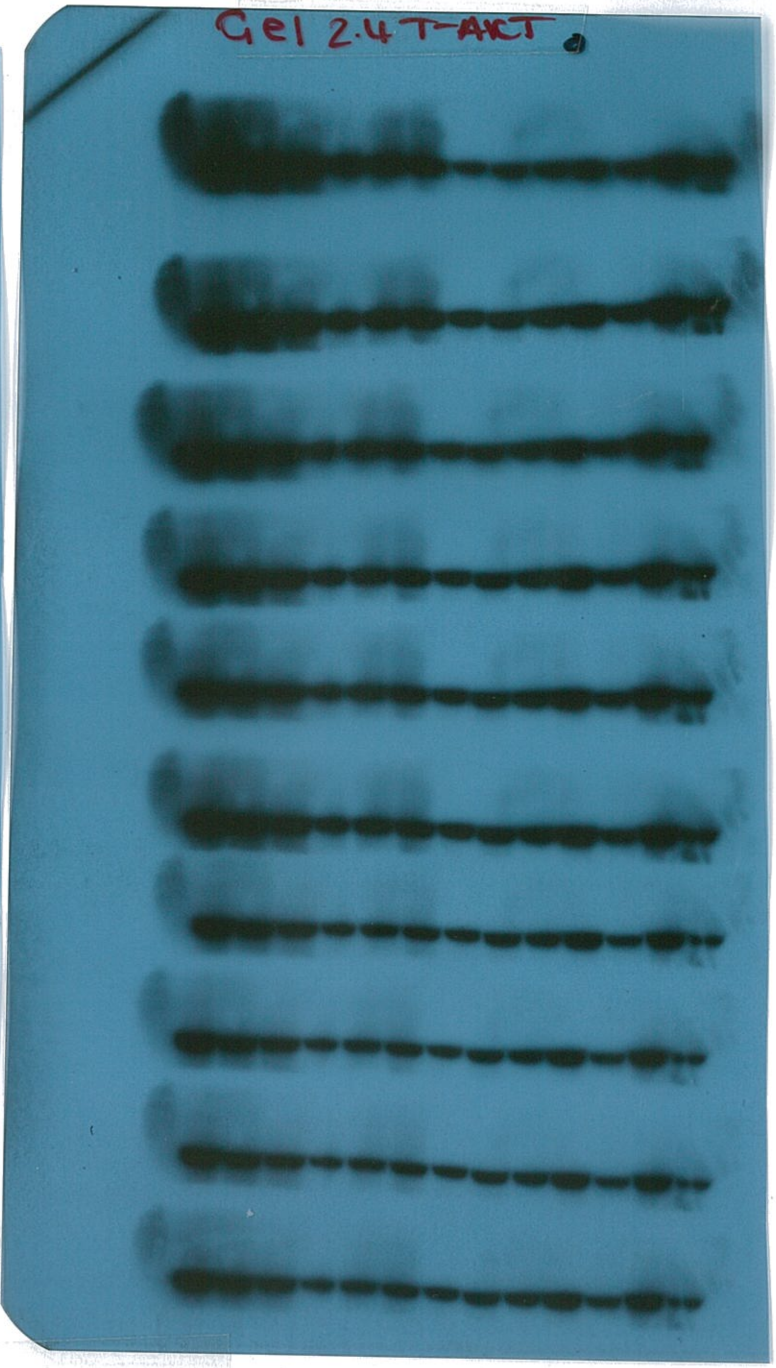
Gel 2.4 T-AKT

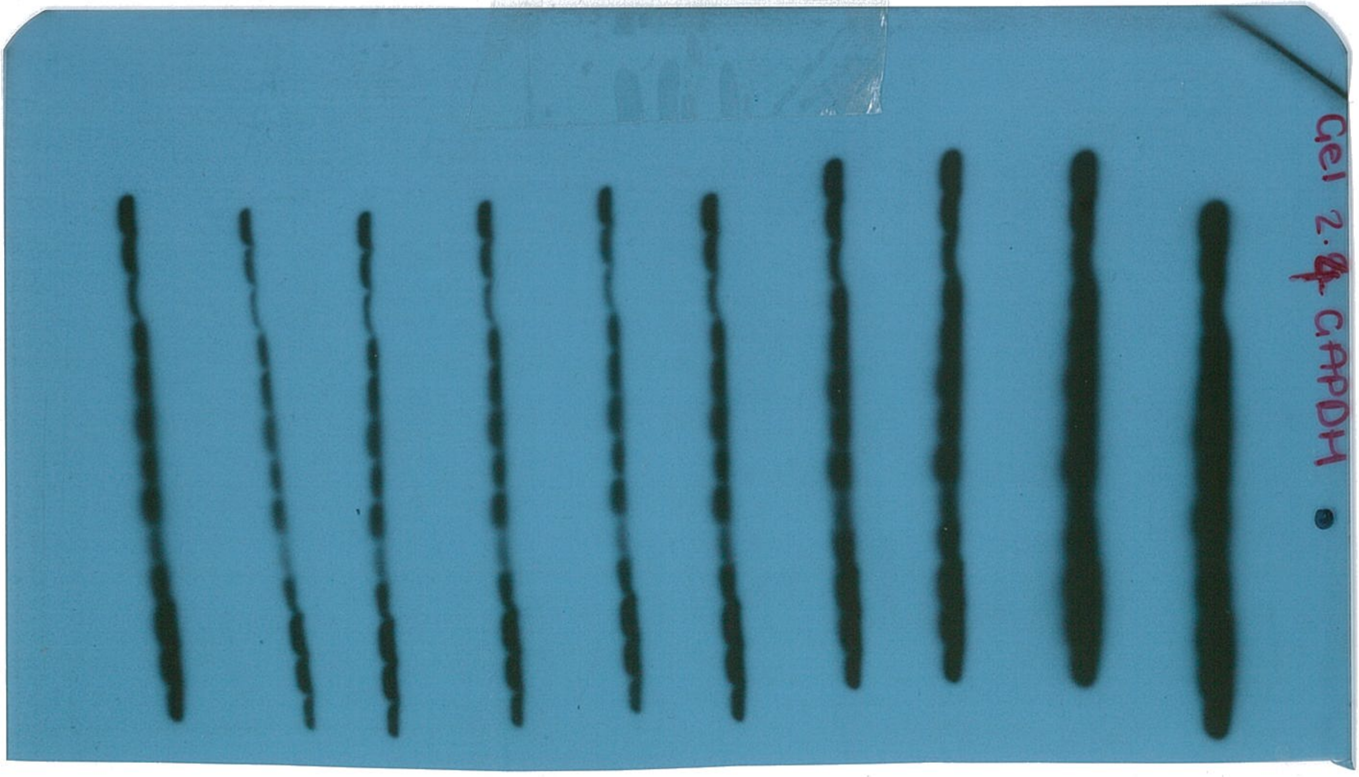
Gel 2.4 GAPDH

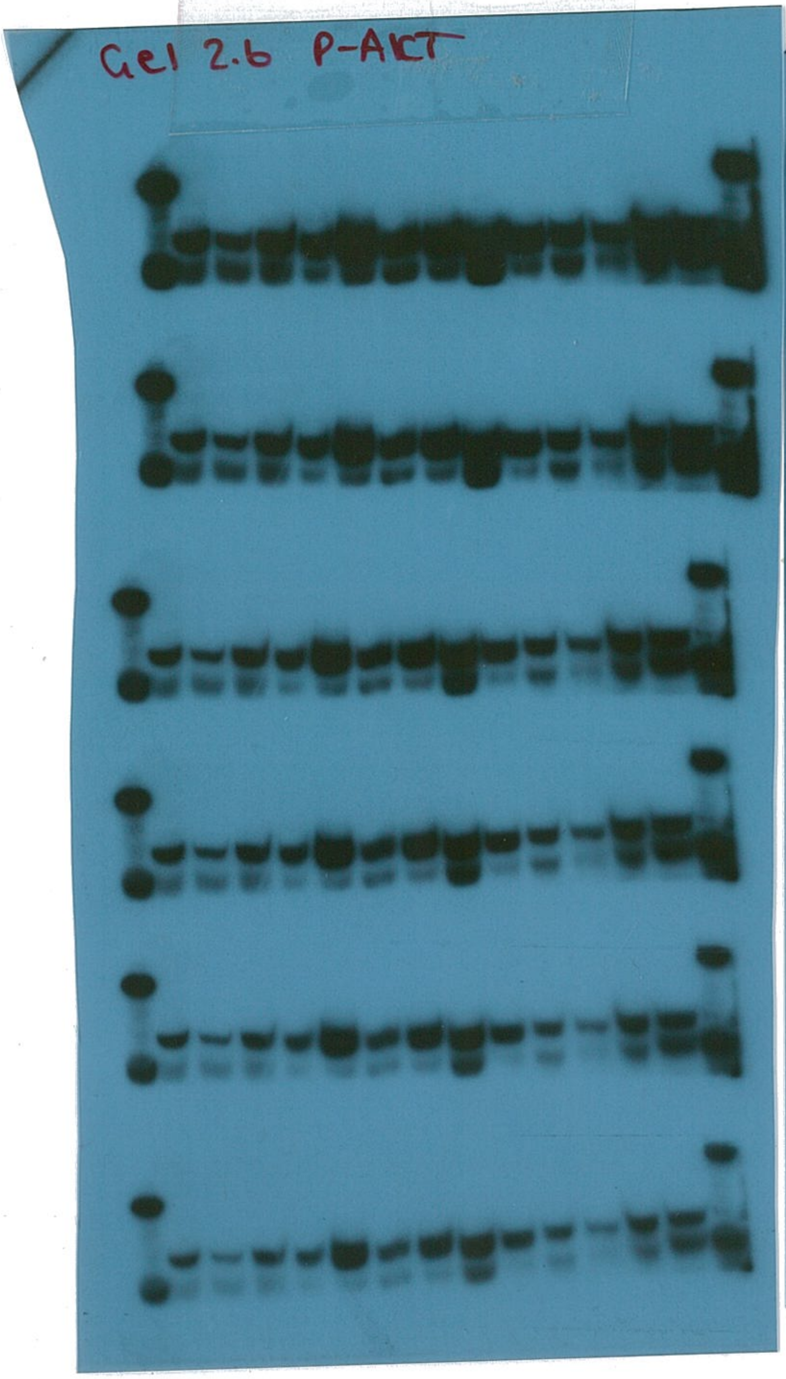
Gel 2.6 P-AKT

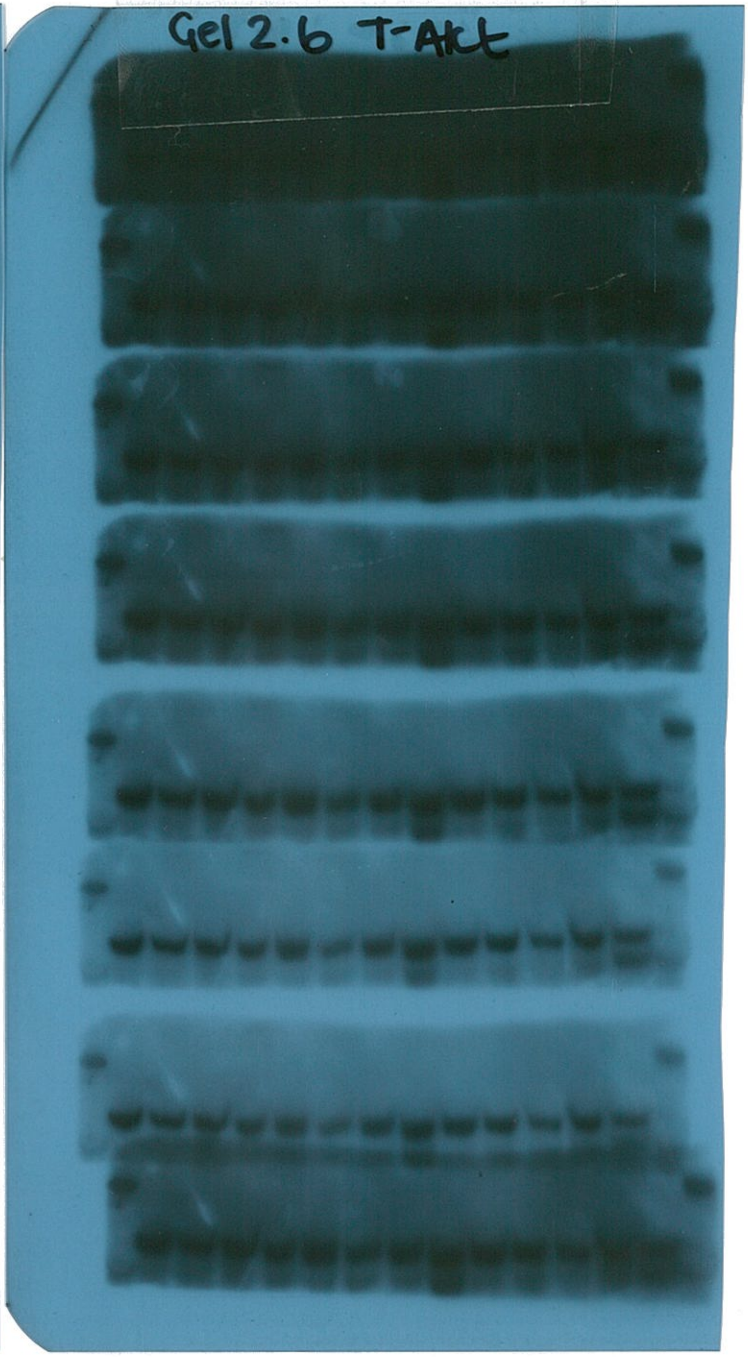
Gel 2.6 T-Akt

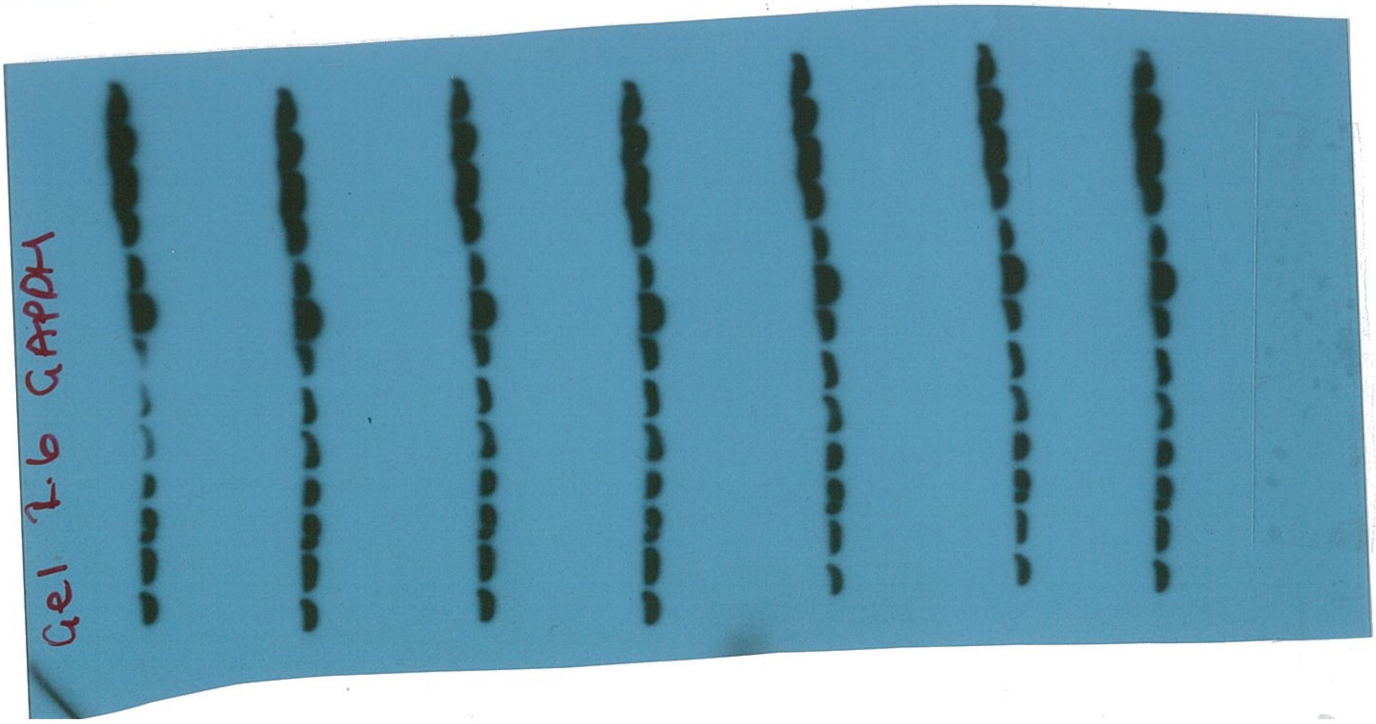
Gel 2.6 GAPDH
