# Supplementary material for: NOTCH and AKT Signalling Interact to Drive Mammary Tumour Heterogeneity
Source: Cancers (Basel). 2023 Aug 29;15(17):4324. doi: 10.3390/cancers15174324 (PMC10486941; doi:10.3390/cancers15174324)
Supplement: Supplementary file 1 [file cancers-15-04324-s001.zip › Supplementary Information_revision 1.pdf]

Supplementary Information

Supplementary Figure S1. Additional examples of ΔNp63 positive cells in mammary ducts adjacent to tumours (upper row, both examples from MS1520-1, *Pten*$^{f/f}$ *p53*$^{f/+}$ *Notch* wild type; bottom left, MS1542-4, *Pten*$^{f/f}$ *p53*$^{f/+}$ *Notch 1*$^{f/+}$; bottom left, MS1329-1, *Pten*$^{f/f}$ *p53*$^{f/+}$ *Notch2*$^{f/f}$). White arrows indicate examples of positive cells with elongated nuclei lying flat against the duct at the outer periphery, consistent with myoepithelial cells. Black arrows indicate examples of cuboidal to columnar cells in a suprabasal or luminal position, consistent with a luminal epithelial cell identity but showing atypical p63 staining. Bar = 50 um.

Supplementary Figure S2. Scans of original western blot films and exposures used for quantitation to generate Figure 5. Blots are grouped as 'Gel Sets' 1 – 4, each of which corresponds to a set of triplicate experiments quantified in Supplementary Table 5. For each Gel Set, the original blots showing multiple exposures have been scanned. The regions which were scanned for quantitation for each replicate are indicated on the first page of each Gel Set. Numbering of the lanes corresponds to the numbering of samples in Supplementary Table 5. Lanes with red numbering were not quantified.

Supplementary Data Table S1. Details of mice used in study. A separate tab is provided from each line giving the mouse ID, dates of birth and death, age, genotype, 'block' (pathology reference number for each tumour) and tumour phenotype diagnosis.

Supplementary Data Table S2. qrtPCR probes.

Supplementary Data Table S3. qrtPCR expression values used to generate Figure 1. $\Delta\text{–}\Delta_{Ct}$ values for *Notch1-4* normalised to β-actin and expressed as mean fold gene expression differences with 95% confidence intervals for triplicate technical repeats over the comparator sample ($CD24^{High}$ $Sca1^{-}$ luminal ER negative stem/progenitor cells from normal C57Bl6 mice). 'Sample' indicates the block number of the tumour as previously published [1, 2]. The genotype and parity status of the animal are provided, as are the diagnosis of the tumour and its estrogen receptor status, all from previous publications.

Supplementary Data Table S4. qrtPCR expression values used to generate Figure 4. $\Delta\text{–}\Delta_{Ct}$ values normalised to β-actin and expressed as mean fold gene expression differences over the comparator sample (tumour 1309-1). Log2 transformed values are provided on a separate tab.

Supplementary Data Table S5. Results of quantitation of AKT activation western blots. Each tab includes the raw image J analysis data, both background readings and band intensity (which are density readings, therefore lower than the background readings as the latter represent how bright a clear piece of film is) from a 'Gel Set'. One Gel Set is the set of triplicate western blots run on a group of samples (see also **Supplementary Figure S1**). Note that for some gels spare samples were loaded into wells but not quantified in that particular gel set. For each sample in each replicate, the background value was subtracted from the band intensity, then total AKT and pAKT values were normalised to GAPDH, then the GAPDH-normalised pAKT was normalised to the GAPDH-normalised total AKT. Each normalised value for AKT activation was then normalised to the common control sample used across all gels and finally the triplicates were combined to get a mean level of AKT activation. The results are summarised on the 'All Sets' tab.
