# Supplementary figures and images for: NOTCH and AKT Signalling Interact to Drive Mammary Tumour Heterogeneity
Source: Cancers (Basel). 2023 Aug 29;15(17):4324. doi: 10.3390/cancers15174324 (PMC10486941; doi:10.3390/cancers15174324)

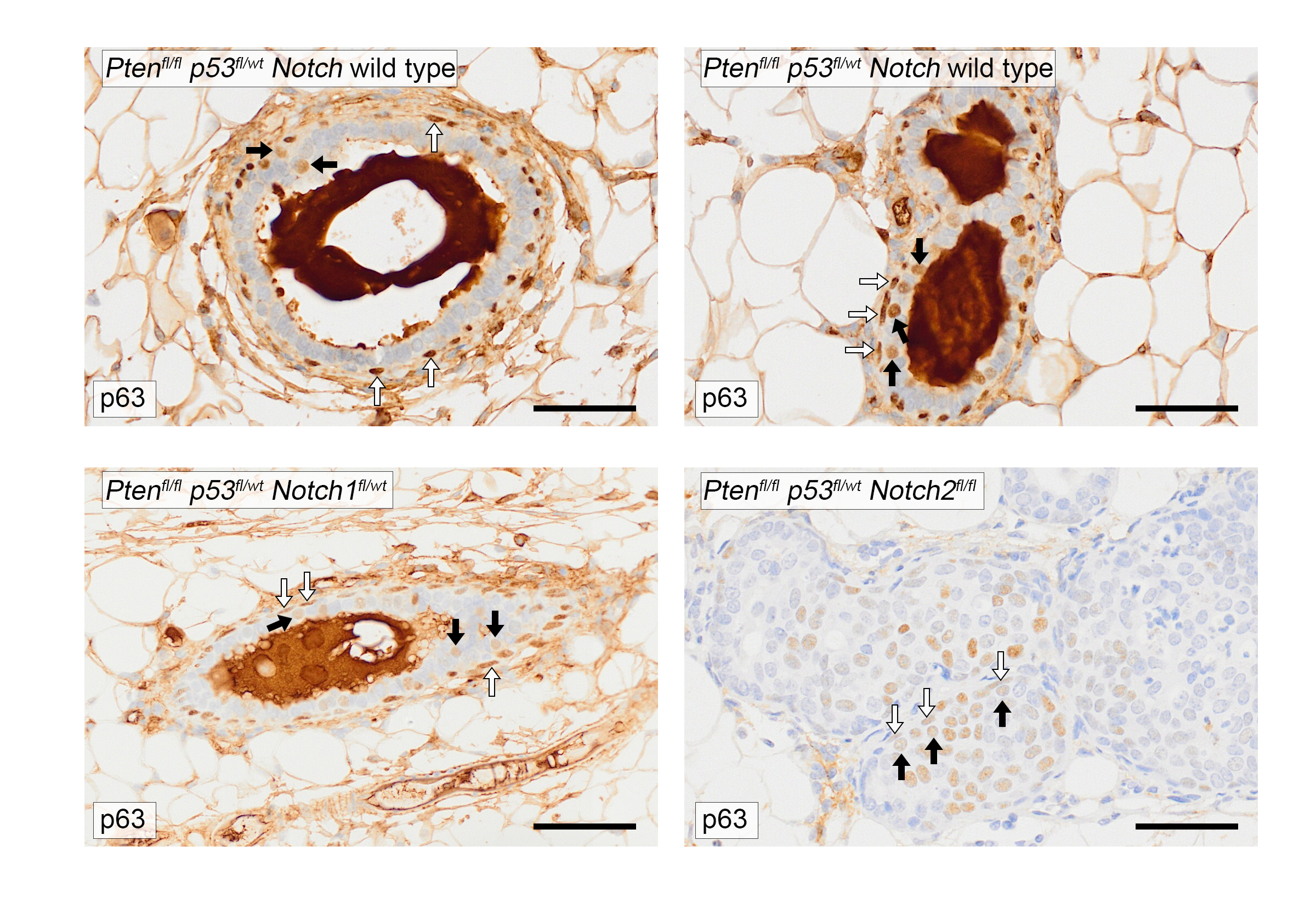

Supplement: Supplementary file 1 [file cancers-15-04324-s001.zip › Supplementary Figure S1_revision 1.tif]
